# Pheochromocytomas and Paragangliomas—Current Management

**DOI:** 10.3390/cancers17061029

**Published:** 2025-03-19

**Authors:** Adam Brewczyński, Agnieszka Kolasińska-Ćwikła, Beata Jabłońska, Lucjan Wyrwicz

**Affiliations:** 1 Oncology and Radiotherapy Department, Maria Skłodowska-Curie National Research Institute of Oncology, 02-034 Warsaw, Poland; agnieszka.kolasinska-cwikla@nio.gov.pl (A.K.-Ć.); lucjan.wyrwicz@nio.gov.pl (L.W.); 2 Department of Digestive Tract Surgery, Medical University of Silesia, 40-752 Katowice, Poland

**Keywords:** pheochromocytoma, paraganglioma, carotid body tumor, jugulo-tympanic paraganglioma, vagal paraganglioma, adrenalectomy, stereotactic radiosurgery, SDHx mutation

## Abstract

Pheochromocytomas and paragangliomas (PPGLs) are infrequent neuroendocrine hypervascular neoplasms arising within different sites of the paraganglion system. They are divided into sympathetic (including pheochromocytomas and extraadrenal paragangliomas) and parasympathetic extraadrenal tumors. These tumors are usually not malignant and grow slowly; about 90% of them are found in the adrenal paraganglia (pheochromocytomas). Extraadrenal tumors are most frequently located in the abdominal cavity (85%), followed by the thoracic cavity (12%), and head and neck (3%). About 25% of PPGLs are related to germline mutations, which are risk factors for multifocal and metastatic disease. In PPGL diagnostics, laboratory, biochemical, and imaging (anatomical and functional) examinations are used. Surgery is the standard management choice for locoregional disease. For patients who are not candidates for surgery and who have stable, not-growing, or slow-growing tumors, active observation or other less invasive techniques (i.e., stereotactic surgery, hypofractionated stereotactic radiotherapy) are considered. In metastatic disease, systemic therapies (tyrosine kinase inhibitors [TKIs], mTORC1 inhibitor everolimus, immunotherapy, cold somatostatin analogs [biotherapy], and radioligand therapy) are used. The prognosis for PPGLs is quite good, and the 5-year survival rate is >90%. The goal of this paper is to review knowledge on the etiopathogenesis, current diagnostics, and therapy for PPGL patients. Our paper is particularly focused on the current management of PPGLs.

## Simple Summary

Pheochromocytomas and paragangliomas (PPGLs) are infrequent neuroendocrine hypervascular neoplasms that arise at different sites of the paraganglion system. They are divided into sympathetic (including pheochromocytomas and extraadrenal paragangliomas) and parasympathetic extraadrenal tumors. The extraadrenal tumors are located in the abdominal cavity (85%), thoracic cavity (12%), and head and neck (3%). About 25% of PPGLs are related to germline mutations, which are risk factors for multifocal and metastatic disease. Laboratory tests, as well as anatomical and functional imaging, are used in PPGL diagnostics. The treatment of PPGLs is still in progress; however, surgery remains the first-line management option for locoregional disease. For patients who are not candidates for surgical treatment and have stable, not-growing, or slow-growing tumors, active observation or other less invasive techniques (i.e., stereotactic surgery, hypofractionated stereotactic radiotherapy) are considered. In advanced and metastatic PPGLs, various types of systemic therapy are used. This review presents current knowledge regarding the etiopathogenesis, current diagnostics, and therapy for PPGL patients. Our paper is particularly focused on the current management of PPGLs.

## 1. Introduction

Pheochromocytomas and paragangliomas (PPGLs) are infrequent neuroendocrine hypervascular neoplasms arising within different sites of the paraganglion system. These tumors are most frequently not malignant, they grow slowly, and about 90% of them are located in the adrenal paraganglia (pheochromocytomas) [1]. The extraadrenal tumors are located in the abdominal cavity (85%), thoracic cavity (12%), and head and neck (3%) [1]. The “paraganglion system” was a description introduced by Mascorro and Yates for a group of neuroectoderm-derived chromaffin cells in extraadrenal locations [1,2].

Head and neck paragangliomas (HNPGLs) develop from neural crest-derived cells present along the jugular foramen, middle ear, carotid bifurcation and vagal nerve. Carotid body paragangliomas (CBPs)/carotid body tumors (CBTs) are the most frequent (60%) HNPGLs, followed by jugular foramen and vagal PGLs (VPs). The other less frequently reported PGL locations are as follows: nasal cavity, larynx, orbit, trachea, aortic body, lung, and mediastinum [3,4]. HNPGLs constitute 0.6% of all cancers of the head and neck. The frequency of HNPGLs is not high (about 1–8 cases per million patients) [3,5,6,7]. Up to 95% of HNPGLs are nonsecretory. HNPGLs are frequently related to succinate dehydrogenase (SDHx) genetic mutation. SDHx germline mutations are found in about 40% of HNPGLs [3,8,9,10,11,12].

PPGLs are manifested by various signs, depending on their anatomic site. PPGLs are manifested frequently by three typical symptoms, including diaphoresis, headache, and palpitations as well as hypertension [10]. It should be added that asymptomatic patients are observed more frequently now than in the past [10]. CBPs arising usually in the carotid artery bifurcation and vagal PGL growing from the inferior vagal ganglion typically manifest as a neck mass, cough, hoarseness, and dysphagia. Tympanic PGLs (TPs) arising from the tympanic plexus of Arnold’s and Jacobson’s nerve or the jugular bulb as jugular PGLs typically manifest as otalgia, pulsative tinnitus, hearing loss, or lower cranial nerve deficits caused by their compression [3,13,14,15,16].

The treatment of PPGLs is still in progress, but surgery is the first-line management option. Recently, the frequency of various methods of nonsurgical less invasive management has been increased [3].

The aim of this paper is to present the literature concerning the current management of PPGLs.

## 2. The Strategy of the Literature Research

In this narrative review, the PubMed database was reviewed. The search terms and mesh heading were as follows: “pheochromocytoma”, or “paraganglioma”, or “head and neck paraganglioma”, or “carotid body paraganglioma” or “tympano-jugular paraganglioma”, or “vagal nerve paraganglioma”, or “pheochromocytoma and surgery” or “paraganglioma and surgery”, or “pheochromocytoma and treatment”. Selected full-text English language articles on the selected topics were read and analyzed. All recent (written after 2010) meta-analyses regarding current PPGL management were included and shortly summarized in our review.

## 3. Pheochromocytomas and Paragangliomas According to World Health Organization and American Joint Committee on Cancer Classifications

There are two different classifications of PPGLs according to the World Health Organization (WHO) fifth edition (2022) cancer classification and the American Joint Committee on Cancer (AJCC) eighth edition (2017) cancer classification. In the WHO classification, a tumor overview is indicated as the most important method, while in AJCC, TNM classification is considered the most important method. Both classifications define pheochromocytomas (PCCs)/adrenal PGLs as arising from the adrenal medulla and all other PGLs as extraadrenal PGLs (EAPGLs) [17,18,19].

### 3.1. WHO Classification

**Figure 1:**
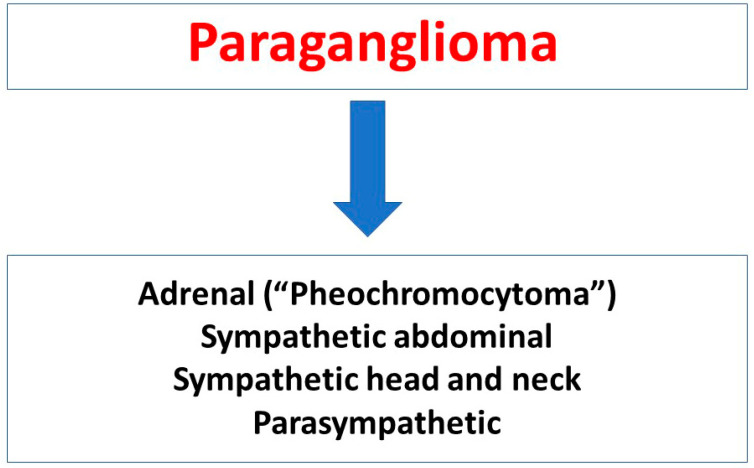
Division of paragangliomas according to the current WHO classification (2022).

In the WHO classification, pheochromocytoma (PCC)—adrenal paraganglioma (adrenal PGL), extra-adrenal paraganglioma (EAPGL), and metastatic neoplasms are distinguished. The 2022 WHO classification defines PCCs as adrenal PGLs. Adrenal and extraadrenal PGLs are collectively defined as PPGLs. According to WHO, most parasympathetic PGLs are found in the head and cervix, while most sympathetic PGLs occur along the skull base to the pelvic cavity. The WHO does not recommend use of the terms “benign” and “malignant” for PGLs because metastatic potential is present in all PPGLs. Metastatic PPGLs are diagnosed in patients in whom PPGL tissue is found in non-paraganglia tissue and there are no clear-cut features predicting metastatic behavior. Moreover, some tumors are lethal despite the absence of metastases because of their locoregional invasion involving important anatomical organs [17,18]. The division of PPGLs is presented in Figure 1 [17].

### 3.2. AJCC Classification

The AJCC 8th edition defines TNM staging in all PGLs during initial diagnosis. In AJCC, the WHO terms of localized and metastatic PPGL are not used, while the terms “benign” and “malignant” are proposed. The term “malignant” refers to metastatic disease. It should be noted that according to AJCC, HNPGLs are benign neoplasms and therefore are not involved in TNM. However, metastases are reported in some HNPGLs. According to AJCC, prognostic staging is based on the tumor diameter, anatomic site, and the presence of metastases [17,19].

According to the literature, approximately 25% of PPGLs are malignant (about 10% of adrenal PGLs and approximately 20% of EAPGLs). Distant metastases are reported in the liver, lung, bone, and lymph nodes, and they are present during the initial diagnosis or even 20 years following the primary neoplasm [10]. A 5-year overall survival rate of 50% is noted in patients with metastatic PPGLs. In about 50% of PPGLs with metastases, genetic SDHB mutations are present; however, in many patients, there are no known mutations. Additionally, some patients have clinically aggressive neoplasms where, although there are no metastases, there is deep local invasion into adjacent structures, surgical resection of the tumor is not possible and clinical presentation is secondary to catecholamine hypersecretion [10].

## 4. Histopathology

Macroscopically, HNPGLs are gray white to pink-tan, firm, ovoid viscid tumors. Microscopically, the tumors are built of cells growing in nests, or zellballen (“ball of cells”). The histopathology of metastatic PGLs does not differ from nonmetastatic PGLs. Metastatic PGLs are recognized in patients with distant metastases [17,20].

Chromogranin-A is the most specific PGL marker. It is the most specific marker for all neuroendocrine neoplasms [21,22,23]. GATA3 is another useful PGL biomarker, but it is characterized by a wide (5–95%) variation in expression assessment [21,24]. The mitotic activity is very infrequent (commonly Ki67 is <1/10 hpf [21,25]. Nowadays, there is no specific biomarker for the differentiation of benign and malignant PGL behavior [21,22].

**Figure 2:**
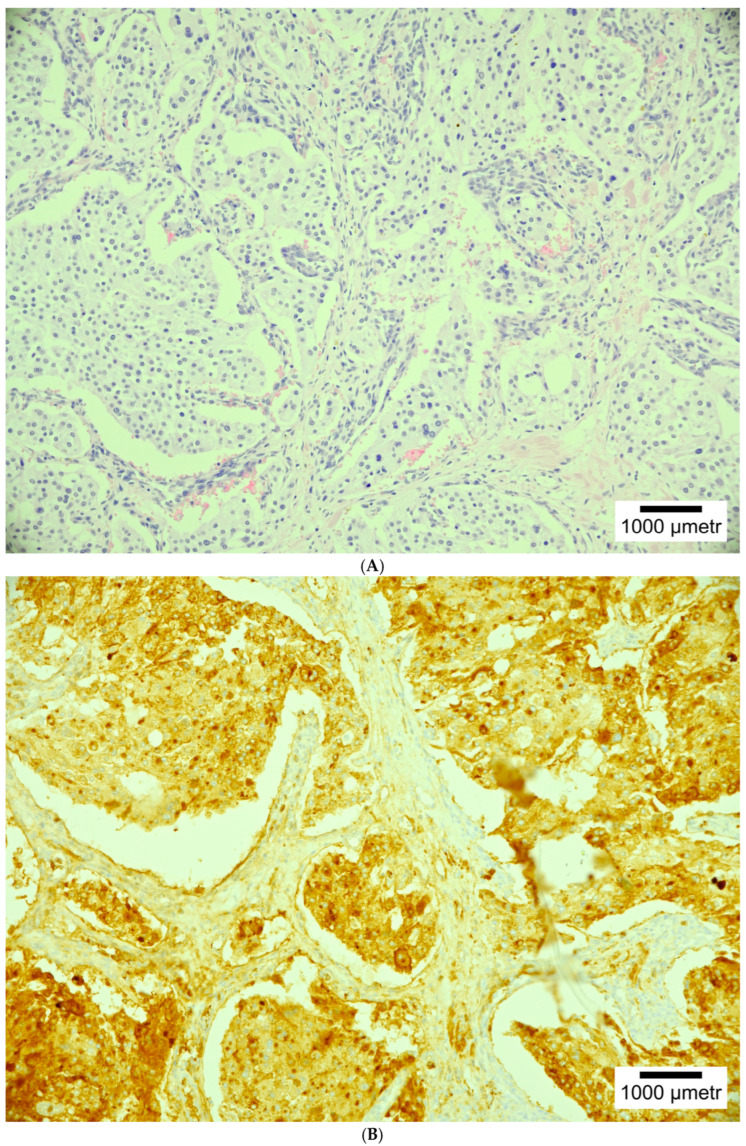

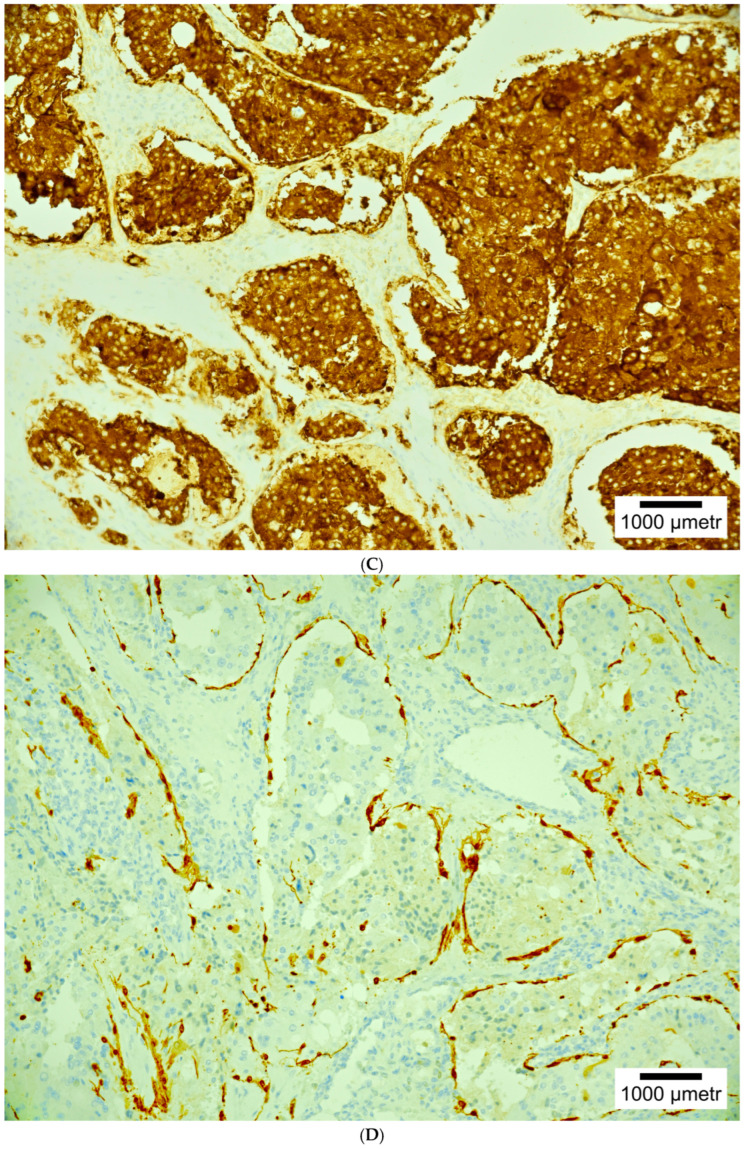

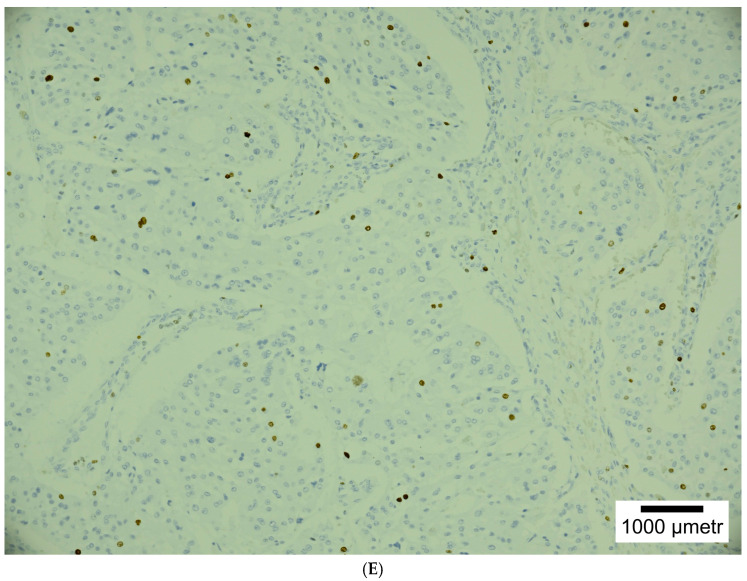
(**A**) Histopathological picture: Hematoxylin and Eosin (H&E) in retroperitoneal paraganglioma in 52-year-old white male patient; (**B**) histopathological picture: Chromogranin A (CgA) (+++) in retroperitoneal paraganglioma in 52-year-old white male patient; (**C**) histopathological picture: Synaptophysin (++) in retroperitoneal paraganglioma in 52-year-old white male patient; (**D**) histopathological picture: S 100—focally positive in retroperitoneal paraganglioma in 52-year-old white male patient; and (**E**) histopathological picture: Ki-67 (2–3%) in retroperitoneal paraganglioma in 52-year-old white male patient.

Prediction of the malignant potential of PPGLs is difficult. According to the literature, a higher risk of malignancy is noted in neoplasms with a size > 4–5 cm, that are secreting methoxytyramine, and are related to germline *SDHB* mutations [10]. There are two pathologic scores for the malignant potential of adrenal PPGLs: the Adrenal Gland Scaled Score (PASS) and GAPP (Grading system for Adrenal Pheochromocytoma and Paraganglioma), which are presented and compared in Table 1 [10]. Various histopathological pictures are presented in Figure 2A–E.

## 5. Epidemiology, Clinical Presentation, Secretory Function, Genetics and Molecular Clusters

### 5.1. Epidemiology

PGLs are reported at any age; however, most PGLs are presented between 30–60 years of age. Germline and sporadic tumors can be distinguished. Hereditary PGLs account for up to 40% of patients [17,26,27,28]. Germline mutations are noted in up to 79% of PPGL patients with a positive family history of non-symptomatic PPGLs and 54% in HNPGL patients [28]. Hereditary PGLs manifest at earlier ages, including in children [17,29,30,31]. Regarding gender, according to some authors, a similar frequency of PGLs in males and females is reported. According to other authors, PGLs are reported more frequently in women [17,30,32]. It has been shown that HNPGLs present at older ages, predominantly in females [3,17,33].

### 5.2. Clinical Presentation

Sympathetic and parasympathetic PGLs can be distinguished. Parasympathetic HNPGLs are the most common, while sympathetic HNPGLs are reported rarely. Most parasympathetic PGLs are nonfunctional [17]. Functional sympathetic PGLs are manifested by clinical symptoms related to tumor secretion; these are presented in Table 2 [10].

As typically nonfunctional tumors, HNPGLs most frequently present as a painless lesion. A carotid body tumor can be detected secondary to squeezing of the carotid arteries. Lower cranial deficits, manifesting as a hoarse voice, dysphagia, shoulder weakness, or Horner syndrome, can be secondary to skull base invasion or squeezing of the cranial nerves. Hearing loss, pulsatile tinnitus, aural fullness, vertigo, and headache can be manifestations of skull base or middle-ear invasion. The secretion of noradrenaline is reported in about 5% of HNPGLs; signs of sympathetic hypersecretion, including tachycardia, hypertension, and sweating, are then reported [5,17,23,33,34,35,36,37,38,39,40,41,42,43,44,45].

### 5.3. Secretory Function

As mentioned above, HNPGLs typically arise from the nonsecretory head and neck parasympathetic ganglia. Therefore, they are usually nonfunctional. Catecholamines are secreted in rare cases. In the adrenal medulla, conversion of noradrenaline to adrenaline is performed; therefore, a difference in pheochromocytomas can be seen. The secretion of adrenaline is not reported in HNPGLs, while noradrenaline is produced very rarely (5% of HNPGLs). Therefore, in HNPGL patients with high noradrenaline/adrenaline levels, a synchronous pheochromocytoma or secretory PGL should be considered and the treatment of a synchronous functioning tumor prior to the management of HNPGL is recommended [4,23,46,47,48,49].

The kidney is the main excretion pathway of catecholamines. Therefore, laboratory assessment of the presence of urine metabolites is useful in HNPGL diagnostics. Most frequently, metanephrine, normetanephrine, and vanillylmandelic acid are assessed from 24 h urine tests. High-performance liquid phase chromatography for plasma-free metanephrine or normetanephrine (specificity 96.1%, sensitivity 94.1%) is preferred in HNPGL diagnostics. In cases of dopamine-secreting HNPGLs, assessment of the 3-methoxythyramine levels in plasma and urine is the best diagnostic tool [21,50,51]. According to recent guidelines, failure in the identification and treatment of high catecholamine concentrations leads to significant morbidity and mortality. Therefore, determination of the functioning HNPGL behavior is currently mandatory before the start of any other management options [21,51]. For measurements of plasma-free metanephrines, patients are required to be in a supine position [52].

According to the literature, in diagnostics, both 24 h urine-fractionated and plasma-free metanephrines are characterized by 90% sensitivity for PPGLs. Plasma metanephrines are the optimal screening test. Because of frequent false-positive results in plasma and urine catecholamines in laboratory tests, these tests are not routine during initial screening except for patients with a succinate dehydrogenase (*SDHx*) mutation, as there are dopamine-only secreting tumors in these patients [10].

### 5.4. Genetics and Molecular Clusters

Germline mutations are reported in about one third of HNPGLs. Hereditary HNPGLs are associated with the mutations of 10 different genes. Mutations of the genes succinate dehydrogenase subunit D (SDHD), succinate dehydrogenase complex assembly factor 2 gene (SDHAF2), succinate dehydrogenase subunit C (SDHC), and succinate dehydrogenase subunit B (SDHB) are reported in PGL syndromes 1, 2, 3, and 4. Succinate dehydrogenase subunit A (SDHA), von Hippel–Lindau (VHL), and transmembrane protein 127 (TMEM127) gene mutations are also related to HNPGLs. HNPGLs in patients with rearranged during transfection (RET), neurofibromatosis type 1 (NF1), and MYC-associated factor X (MAX) gene mutations are noted rarely [53]. SDHx germline mutations are reported in up to 40% of HNPGLs [3,53,54].

The familial presence of HNPGLs has been reported in the world literature. In 1933, two sisters with CBTs were described by Chase [53,55]. Also, in 1933, Goekoop reported jugulo-tympanic PGLs in three sisters [53,56], and familial vagal PGLs were reported in 1956 [53,57]. Four hereditary PGL syndromes (PGL1–4) have been distinguished. In 2000, Baysal et al. [58] first described hereditary SDHD gene mutations gene in PGL1 syndrome [53,58]. The associations of PGL3 syndrome with SDHC, and PGL4 syndrome with SDHB mutations were found [53,59,60]. In 2009, mutations of the succinate dehydrogenase complex assembly factor 2 (SDHAF2) gene (SDH5), were described in PGL2 syndrome [53,61,62]. Generally, inherent genetic mutations related to PPGLs can be classified into two major clusters depending on their gene expression profile: Cluster 1 or the angiogenic cluster genes are involved with the pseudohypoxia and they are as follows: PHD2, von Hippel–Lindau (VHL), succinate dehydrogenase complex (SDHx), Isocitrate Dehydrogenase (IDH), HIF2A, malate dehydrogenase 2 (MDH2) and fumarate hydratase (FH) mutated PPGL. Cluster 2 or kinase signaling cluster involve genetic mutations related to kinase signaling, including PI3Kinase/AKT and the mTOR pathway. Cluster 1 is divided into subcluster 1A and 1B. Cluster 1A involves PPGL related to SDHx and FH, Cluster 1B involves tumors with endothelial PAS domain protein 1 (HIF2A) and VHL, respectively. Cluster 2 involves the following mutations: kinesin family member1B (KIF1B), the rearranged during transfection (RET) proto-oncogene, neurofibromin 1 (NF1), transmembrane protein 127 (TMEM 127), MYC-associated factor X (MAX), and menin (MEN1). Cluster 2 involves groups 2A, 2B, 2C and 2D. Group 2A contains RET, MAX, NF1 and TMEM127 mutated tumors, while groups 2B and 2C are sporadic tumors. The genes in cluster 2, RET/NF1/TMEM127/MAX/KIF1Bβ, are connected to oncogenic kinase signaling pathways. Additionally, other gene mutations (*GDNF*, *H-ras*, *K-ras*, *GNAS*, *CDKN2A* (*p16*), *p53*, *BAP1*, *BRCA1&2*, *ATRX*, *KMT2D*, HIF2A, EPAS1, H3F3A, EGLN2, MDH2, ATRX, CSDE1, MAML3, IRP1, SLC25A11, and DLST mutations) have been described in PPGLs [63]. In addition, a kinesin family member 1B (KIF1B) missense mutation in pheochromocytomas has been identified. KIF1B functions as a tumor suppressor that is required for apoptosis [53]. In addition, a prolyl hydroxylase domain 2 (PHD2) mutation in a patient with erythrocytosis and recurrent PGL has been described [53]. Different PGL locations in hereditary syndromes are presented in Table 1 [17,53,64]. In addition, non-KIT/PDGFRA gastrointestinal stromal tumors (GISTs) can be related to mutations in the SDHB, SDHC and SDHD genes related to PGL in Carney–Stratakis syndrome. Carney–Stratakis syndrome (Carney dyad) is an autosomal dominant syndrome associated with mutations in SDHB, SDHC and SDHD. This syndrome is reported in both genders at a mean age of 23 years. It is characterized by PGLs and GISTs, but not pulmonary chondroma, in contrast to the Carney triad. The Carney triad is a rare disorder reported in young women at a mean age of 20 years. The classic Carney triad includes extraadrenal sympathetic PGLs, GISTs, and pulmonary chondroma. Adrenal cortical adenoma, esophageal leiomyoma, and adrenal PGLs were also reported in this syndrome. Genetic mutations have not been reported so far in hereditary PGLs in the Carney triad. A case of growth hormone-secreting pituitary adenoma in a patient with PGL1 syndrome was described. Medullary thyroid carcinoma is the most typical clinical manifestation of the disease. Von Hippel–Lindau (VHL) disease is an autosomal dominant, predisposing to renal neoplasms and clear cell carcinoma, pancreatic serous cystic neoplasms (SCNs), and neuroendocrine neoplasms, as well as hemangioblastoma of the eye and central nervous system. For NF1 (neurofibromatosis), multiple neurofibromas, café-au-lait spots, Lisch nodules of the iris and other infrequent diseases are typical [53,64]. In PGL syndromes, patients with HNPGLs and PCCs are significantly younger compared to patients with sporadic paraganglia tumors [53,63]. MEN2 is a well-known autosomal dominant syndrome caused by activating germline mutations of the *RET* gene. MEN2 is subdivided into MEN2A and MEN2B. MEN2A is associated with a classic tumor triad: in 90% percent of mutations with MEN2A, medullary thyroid carcinoma occurs; in 50%, adrenal PGL occurs; and in 20–30% parathyroid hyperplasia occurs. Patients with MEN2B also present with a similar percentage of medullary thyroid carcinoma and adrenal PGL, but do not manifest parathyroid disease. Adrenal PGLs arising as part of MEN2 syndromes are frequently associated with adjacent hyperplasia of the adrenal medulla [52]. Reed’s syndrome (RS) is an autosomal dominant disease characterized by uterus and skin leiomyomas. In some RS patients, renal cell carcinomas (RCC) are reported. RS is caused by mutations in the fumarate hydratase (FH) gene [65]. Pacak–Zhuang syndrome (PZS) of unknown inheritance is associated with an EPAS1 gene mutation and it is manifested by polycythemia, somatostatinoma, retinal abnormalities, and cysts [52]. A mutation of the KIF1B gene is predisposed to developing ganglioneuroma, leiomyosarcoma, pulmonary adenocarcinoma, and neuroblastoma [52]. Pheochromocytoma and paraganglioma locations in hereditary syndromes are presented in Table 3.

Several molecular clusters have been distinguished in PGLs: pseudohypoxia, kinase signaling, and Wnt-altered clusters. In HNPGLs, the pseudohypoxia cluster is the most common [17]. The kinase-signaling cluster is usually found in adrenergic adrenal PGLs that can be bilateral and adrenergic PGLs, all with longer metastatic-free survival. In this cluster, hypermethylation is absent, in contrast to the pseudohypoxia cluster. The Wnt-altered cluster is related to sporadic adrenal PGL and higher norepinephrine secretion. Worse prognosis, metastatic disease, and higher Ki-67 are reported for tumors in this cluster [17]. The associations between biochemical profiles and specific genetic mutations are presented in Table 4 [17].

## 6. Imaging

Various radiological examinations, such as ultrasound (US), computed tomography (CT) and CT angiography, magnetic resonance imaging (MRI) and MRI angiography, conventional arteriography and nuclear functional imaging, including somatostatin receptor imaging, [^18^F]-FDOPA positron emission tomography (PET), and single-photon emission computed tomography (SPECT), are performed in PPGL diagnostics [17]. Abnormal laboratory results (increased metanephrine and catecholamine secretion) or neck tumors are common indications for radiological diagnostics [11,21,63,65,66,67,68].

### 6.1. Anatomical Imaging

Ultrasound is the first-line imaging technique for neck tumors. In US, PGLs are clearly demarcated, solid tumors with a heterogeneous hypoechoic structure and echo-free dilated vessels [21,67]. In color-flow Doppler ultrasound, the hypervascular tumor is demonstrated. The US shows a small tumor in the lateral part of the neck; however, the use of US in diagnostics of high vagal tumors is limited [67]. In addition, this examination does not show tumor details. Therefore, HNPGL detection in US requires further detailed radiological investigations, including CT and MRI [21].

Contrast-enhanced CT (CECT) is very good imaging for anatomic tumor descriptions, and it enables tumors of sizes < 1 cm to be shown. CT is useful for the assessment of skull base invasion and the detection of small PGL tympanicum and jugulo-tympanicum. Homogeneous enhancement at CECT is typical for HNPGLs. CECT is frequently performed as a first-line radiological investigation in patients with a neck mass due to its excellent spatial resolution and ability to detect small tumors and bony invasions [17]. CT angiography is very useful for preoperative vascular assessment, because it perfectly shows anatomical tumor relationships to the internal carotid artery, ipsilateral and contralateral venous return as well as other possible tumor locations. In addition, CECT can also show calcifications within the tumor [67].

Contrast-enhanced MRI is frequently the imaging technique of choice for the detection and assessment of HNPGLs (sensitivity 90–95%, specificity 92–99%) [17]. Contrast-enhanced MRI accompanied by contrast-enhanced MR angiography (MRA) is better at the detection of smaller HNPGLs, compared to contrast-enhanced MRI alone. Currently, MRI has become the reference PGL imaging choice [67]. In T1-weighted sequences, the PGL is solid and clearly demarcated with an isosignal, while in T2-weighted sequences, a high-intensity signal is present. The typical sign is a salt-and-pepper appearance, i.e., punctate or serpiginous low-intensity signals corresponding to rapid flow vessels (pepper) within a global high-intensity signal (salt) corresponding to the high-intensity signal of the tumor matrix or bleeding zones. This characteristic feature is noted in tumors with sizes > 1 cm, but it is not pathognomonic, as it can also be reported in other hypervascular tumors. After gadolinium injection, contrast enhancement is intense, homogeneous or heterogeneous, depending on the presence of necrotic zones. Gadolinium-enhanced sequences enhance the detection of small tumors. MRI sequences are useful for preoperative vascular assessment [67]. Recently, gadolinium-enhanced 3D MR and 4D MR angiography has been introduced into PGL imaging. Rapid and intense contrast enhancement of the lesion at the early arterial phase and early venous return are typical for PGL. This sequence is useful for the assessment of multiple tumors [67].

### 6.2. Functional Imaging

Functional molecular imaging examinations include somatostatin receptor scintigraphy (SRS) and [^18^F]-FDOPA positron emission tomography (PET). The functional imaging is characterized by a very high specificity (up to 100%). It allows for whole body investigation, which is very useful for the detection of multifocal tumors [67].

OctreoScan^®^ is an ^111^Indium-labelled somatostatin analogue preferentially binding to somatostatin receptor type 2 (SST2), most intensely expressed by PGLs. SRS is performed with a gamma camera generally connected to an X-ray scanner (hybrid camera). Acquisitions are performed during the first 48 h (usually within the first 24 h). SRS sensitivity is >90% for tumors of size > 1 cm, but much lower for small tumors [67].

[^18^F]-FDG PET plays an important role in the detection of multifocal tumors, depending on the tumor location, tumor differentiation, and the patient’s genetic status. Although this investigation is not specific for neuroendocrine tumors, [^18^F]-FDG PET is very good for the detection of pheochromocytomas (sensitivity 80–100%). [^18^F]-FDG PET is also very sensitive for the detection of metastatic disease in patients with the presence of SDHB mutations [67].

In addition to expressing norepinephrine transporter (NET), neuroendocrine tumors bind and decarboxylate amino acids such as dihydroxyphenylalanine (DOPA). It has been shown that [^18^F]-FDOPA PET has an excellent sensitivity for HNPGL (>90%). According to numerous authors, [^18^F]-FDOPA PET is an excellent first-line examination for the detection of multifocal HNPGLs (sensitivity up to 100%). It should be added that the detection of small tympano-jugular PGLs not visible on morphological imaging is possible using [^18^F]-FDOPA PET [67].

Gallium-68 (^68^Ga)-labelled somatostatin analogues (SSAs) are useful in PPGL diagnostics and the selection of patients for peptide receptor radionuclide therapy, as a potential alternative or complement to the traditional theranostic approach with iodine-123 (^123^I)/iodine-131 (^131^I)-labelled meta-iodobenzylguanidine [66]. Recently, peptide receptor radionuclide therapy (PRRT) with lutetium-177 (^177^Lu)-labelled DOTA-Tyr3-octreotate (DOTATATE; oxodotreotide) or other yttrium-90 (^90^Y)- or ^177^Lu-labelled somatostatin analogues (SSAs) have been introduced in the management of inoperable/metastatic PPGLs. Nuclear imaging (PET or SPECT) is valuable in planning targeted radionuclide therapy with iodine-131 (^131^I)-labelled meta-iodobenzylguanidine (MIBG) or PRRT with [^177^Lu]-labelled DOTATATE or other related agents [65].

MIBG is an iodinated guanidine analogue that is structurally similar to norepinephrine. Normal uptake of [^123^I]MIBG is reported in the myocardium, salivary glands, lacrimal glands, thyroid gland, liver, lungs, adrenal glands (in up to 80% of cases), bowel, and uterus (during menstruation). [^123^I]MIBG uptake in the adrenal glands is defined as normal if it is mild (lower than, or equal to, liver uptake) and symmetrical, and if the adrenal glands are not enlarged in CT. Increased or asymmetrical uptake of [^123/131^I]MIBG accompanied by the presence of an enlarged adrenal gland is defined as abnormal and frequently indicates PCC. Any extra-adrenal abnormal [^123/131^I]MIBG uptake that is focal and cannot be confirmed as having a normal physiological distribution is described as abnormal [65]. MIBG is radiolabelled with ^123^I or ^131^I. [^123^I]MIBG scintigraphy is strongly preferred over [^131^I]MIBG scintigraphy due to the superior quality images, lower radiation burden of ^123^I, and easier performance of SPECT with ^123^I, and the shorter time between injection and imaging in the use of [^123^I]MIBG scintigraphy (24 h) compared to [^131^I]MIBG scintigraphy (48–72 h) [65].

In HNPGLs, [^18^F]FDOPA and [^68^Ga]DOTA-SSAs are the most sensitive radiopharmaceuticals for PET imaging in sporadic cases. In patients with *SDHx*-related PPGLs, [^68^Ga]DOTA-SSA PET/CT is useful for the detection of very small lesions that cannot be found in [^18^F]FDOPA PET/CT. If [^18^F]FDOPA or [^68^Ga]DOTA-SSA PET/CT is not available, SSTR scintigraphy may be used as an alternative. [^18^F]FDG PET is characterized by high sensitivity in the diagnostics of SDHx-related HNPGLs and can be performed with [^18^F]FDOPA PET/CT for the detection of additional thoracic/abdominal PGLs.

In retroperitoneal, extra-adrenal, non-renal masses, the differential diagnostics of PPGLs from neurogenic tumors, lymph node diseases, and mesenchymal tumors is required. ^18^F-FDOPA and [^68^Ga]DOTA-SSAs are more specific than [^18^F]FDG and can identify more lesions. Therefore, [^68^Ga]DOTA-SSA PET/CT is probably the preferred imaging investigation, particularly for patients with SDHx mutations [65].

[^18^F]FDOPA is very useful in the detection of metastases in patients with sporadic PPGLs. This imaging is less sensitive in patients with SDHx mutations. [^68^Ga]DOTA-SSA is better than [^18^F]FDOPA, regardless of the genetic status. Thus, it is the optimal imaging choice in metastatic PPGLs. [^18^F]FDOPA PET/CT can be used as second-line imaging in patients without SDHB mutations or with an unknown genetic status. [^18^F]FDG PET/CT are recommended in SDHB-related metastatic PPGLs. [^123^I]MIBG may lead to significant underestimation of metastatic disease that can lead to inappropriate management. [^123^I]MIBG is a theranostic radiopharmaceutical; [^123^I]MIBG imaging can be used to assess whether a patient is a candidate for [^131^I]MIBG therapy [65].

In conclusion, CT or MRI is recommended for the initial assessment of PGL locations, with MRI being preferential in children and pregnant or lactating women because of radiation exposure during CT. CT and MRI are very sensitive for the detection of most catecholamine-producing tumors; however, these investigations are not specific for the identification of tumors such as PCC or extra-adrenal PGLs. These limitations are overcome in functional imaging using [^123^I]-labeled meta-iodobenzylguanidine scintigraphy ([^123^I]-MIBG), which is characterized by a higher specificity compared to that of anatomical imaging. This functional imaging has limited specificity in familial PGL syndromes, malignant PPGLs and extra-adrenal PGLs. Recently, new compounds, including, [^18^F]-fluorodopamine ([^18^F]-FDA), [^18^F]-fluoro-dihydroxyphenylalanine ([^18^F]-FDOPA), and [^18^F]-fluoro-2-deoxy-D-glucose ([^18^F]-FDG), have been used in PET. [^18^F]-FDA PET imaging is superior to [^131^I]-MIBG scintigraphy, especially with regard to malignant tumors. [^18^F]-FDOPA PET imaging is superior to [^123^I]-MIBG scintigraphy in PCC detection. However, the sensitivity of [^18^F]-FDOPA for metastatic PGLs is limited. The uptake of [^18^F]-FDG in PET imaging is present in PCC. [^18^F]-FDG is a good investigation option in the imaging of metastatic adult PPGLs related to SDHB mutations [68].

The precise local staging and identification of multifocal forms (metastatic forms are very rare—less than 5%) are the main problems in HNPGL diagnostics. SRS can be considered as a first-line examination in non-hereditary tumors in patients for whom genetic analysis is negative. Currently, SRS is more frequently replaced by PET [67]. PET is very useful in hereditary PGLs because of a high risk of multifocal tumors. ^68^Ga-labelled somatostatin agonist PET is also the preferred examination. [^18^F]-FDOPA PET can be suboptimal. A very high sensitivity for [^18^F]-FDG PET is described in hereditary tumors. In patients with an unknown genetic status, a family and personal PGL history, multiple tumors and age ≤ 40 years can suggest a hereditary PGL form [67]. Chen et al. [68] recommended the use of functional imaging in patients with all PPGLs, except adrenal PCCs of diameter >5 cm and those related to elevations of plasma and urine metanephrine [68].

In general, a large tumor size (>5 cm for PCCs, >4 cm for PGLs), vascular invasion, extra-adrenal location, and SDHB mutation-related tumors are suggestive of metastatic PPGLs [63].

The differentiation of PGL from other tumors or lymph node involvement, including metastases, is important in the presence of a retroperitoneal extraadrenal nonrenal mass. A biopsy is not always useful, and in some patients, it is not indicated due to a high risk of hypertensive crisis and tachyarrhythmia; therefore, it is recommended if PGL is ruled out in any patient presenting with symptoms and signs of catecholamine secretion. Although specific functional imaging is very helpful in distinguishing PCC/PGL from other tumors, it is usually not performed prior to biochemical tests [66]. In HNPGLs, there are also numerous differential diagnoses, such as lymph node metastasis, neurogenic tumor (schwannoma, neurofibroma, ganglioneuroma), jugular meningioma, internal jugular vein thrombosis, internal carotid artery aneurysm, hemangioma, and vascular malposition. Functional imaging is very useful in differential diagnostics [66].

**Figure 3:**
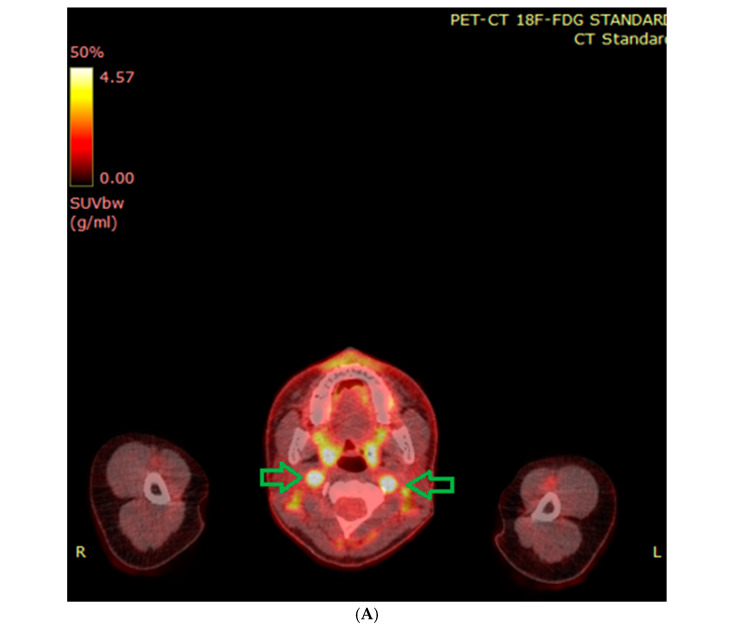

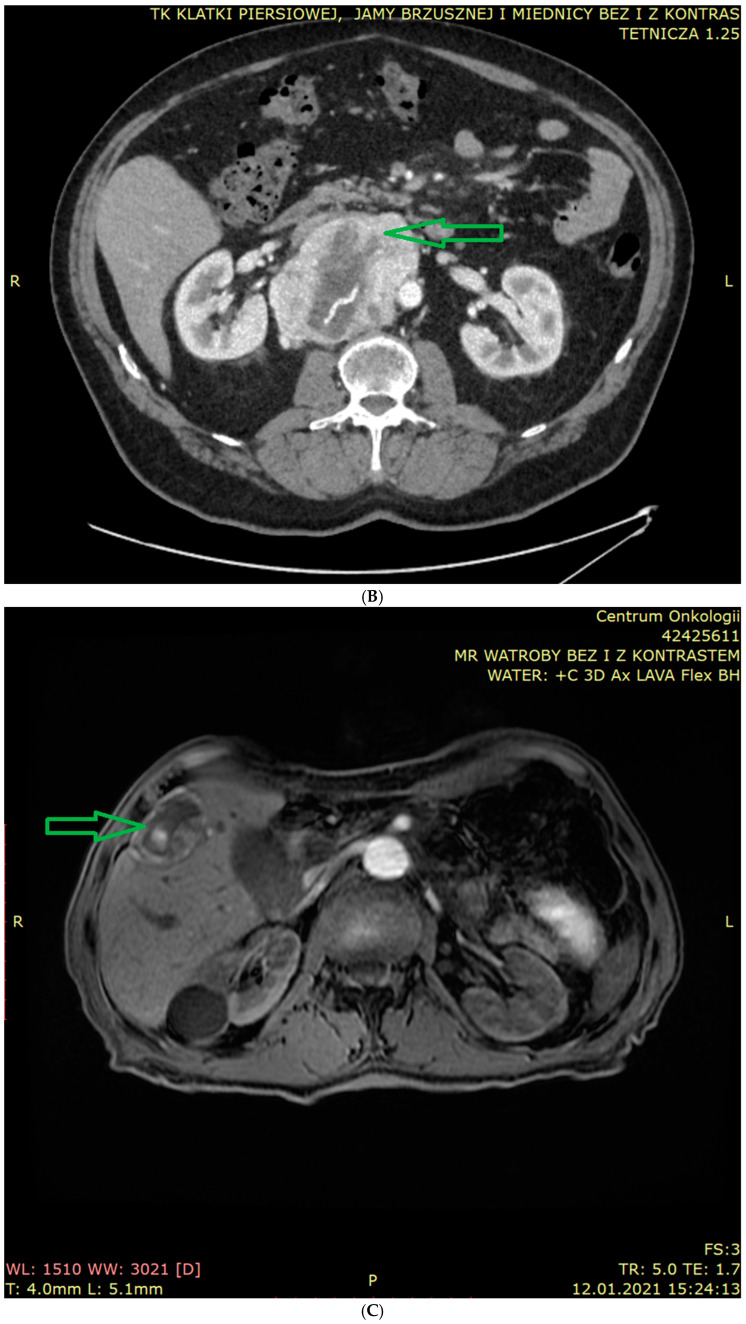
(**A**) PET-CT scan of bilateral neck masses (carotid body tumors, CBT) in a 31-year-old white female patient with familial PPGL1 syndrome—SDHD C11X. (**B**) CT scan of retroperitoneal paraganglioma in a 50-year-old white male patient. (**C**) MRI scan of metastatic pheochromocytoma (liver metastasis) in a 73-year-old white male patient.

Various imaging investigations of PPGLs are presented in Figure 3A–C.

## 7. Current Management

### 7.1. Surgery

Surgery is the standard management practice for patients with locoregional PPGLs. The initial perioperative morbidity and mortality caused mainly by catecholamine secretion was significant (30–45%), but mortality rates (0–2.9%) have been significantly decreased due to current, advanced surgical and medical treatment methods [10].

Preoperative preparation is required for most PPGL patients. Preoperative medical treatment is not needed in patients without any signs (involving normal blood pressure), with normal serum or urinary metanephrines concentrations, regardless of the tumor location, as catecholamines are not produced. Preoperative preparation is also not required in patients in whom tumors produce only dopamine PPGLs (isolated increased serum concentrations of 3-methoxytyramine and absence of hyperadrenergic clinical presentation) [69]. Medical blockade before any surgery or procedure is used for the prevention of a hypertensive crisis in patients with benign or malignant PPGLs. Alpha-adrenergic receptor blockers are commonly used [10].

### 7.2. Abdominal EAPGLs and Pheochromocytomas

Open as well as minimally invasive (laparoscopic and robotic) surgery can be performed on abdominal PPGLs. Currently, minimally invasive surgery is a standard approach for the resection of abdominal PPGLs. According to the literature, lower postoperative morbidity, shorter duration of hospitalization, and lower costs are reported in the laparoscopic approach compared to open surgery [69,70]. Due to the high rate of bilateral PPCs in hereditary cases, partial adrenal resections are indicated for the prevention of complications related to medical adrenal replacement therapy. Partial adrenalectomies in patients with sporadic unilateral PPCs are controversial. It should be added that open surgery is still required for selected patients with locoregionally invasive or malignant PPGLs [68]. According to an international expert consensus statement regarding PPGL management in patients with germline SDHB mutations, open surgical resection is recommended in order to complete dissect vascular and lymphatic structures and in order to decrease the high risk of tumor locoregional recurrence and metastasis in patients with large PPGLs with SDHB mutations [69]. Open surgery is preferable for large tumor sizes (≥6 cm), lymph nodes invasion, large blood vessel invasion, high bleeding risk, and reoperation, while laparoscopic surgery is preferable for small tumor sizes (≤4 cm), clear delineation of surrounding structures, and the absence of blood vessel invasion [69,70]. In patients with SDHB PPGLs, complete tumor removal and avoidance of capsular disruption to decrease the risk of local recurrence and dissemination of tumor cells is most important. The laparoscopic approach is related to a faster recovery compared to open surgery and is better for tumors that are not large and bulky, while an open approach allows for broad exposure and digital tumor palpation (that is not possible in laparoscopic techniques), manual retraction, palpable evaluation of the surrounding tissues, and digital evaluation of thrombus tumor invasion within vessels, if it is needed. Some maneuvers, such as precise lateral clamping of the inferior vena cava, are possible for safe performance during open broad exposition. In the case of an abundantly vascularized tumor, the open approach eliminates the need of conversion, and also saves time, which is very important in the case of life-threatening bleeding [69]. In addition, in the case of large or potentially locally invasive thoracic, paraaortic and pelvic EAPGLs, open surgery allows for hands-on evaluation of the vascular wall invasion and the identification of abnormal lymph nodes. Interpretation of radiological examinations and foreseeing potential vessel involvement is very important for effective perioperative planning [69]. Laparoscopic surgery is not recommended for tumors sized >6 cm due to the high risk of locoregional invasion, recurrence, and metastases [69]. For tumors that are 4–6 cm, an individualized approach selected by a surgeon for a patient is recommended. This recommendation is associated with the fact that a larger size was indicated as an independent risk factor for distant metastases [69,70,71,72,73]. In cases of intraoperative detection of locoregional or vascular invasion during laparoscopic procedure, conversion to an open procedure to allow en bloc resection is required. Both the intraabdominal and retroperitoneal techniques are equally effective in laparoscopic procedures [69]. Adrenal cortical-sparing surgery is indicated for patients with a high risk of bilateral adrenal PGL and a low risk of malignancy, including patients with VHL and MEN2. In patients in whom adrenal cortical function can be preserved, long-term dependence on corticosteroids can be avoided [10]. Cortex-sparing resection is not recommended for patients with SDHB PPCs due to a high risk of local recurrence and/or metastasis [69]. Total adrenalectomy rather than a cortical-sparing procedure, regardless of tumor size, should be performed in these patients. Total adrenalectomy for SDHB PPC is related to a higher risk of locoregional recurrence and metastasis compared to other PPC subtypes [69,72,74,75,76,77,78,79,80]. In a comparison of laparoscopic versus robotic-assisted approaches, in younger patients, with lower body mass index, with complex cardiovascular history, a laparoscopic adrenalectomy is recommended. The disadvantages of robotic-assisted adrenalectomy are as follows: a longer operation duration, higher cost, and an absence of tactile feedback, which could inadvertently increase tumor manipulation and catecholamine release [81,82]. According to the literature, morbidity and mortality rates are similar in both minimally invasive techniques [81,83]. A meta-analysis by Gan et al. [83] comparing robotic-assisted and laparoscopic adrenalectomy in 2985 patients reported in 26 studies showed that the robotic technique was significantly better than conventional laparoscopy for estimated blood loss, duration of hospitalization, and conversion to open procedure, while complications and readmissions were similar in both groups. The duration of operations was comparable in both groups. For pheochromocytoma, robotic adrenalectomy was superior to laparoscopic adrenalectomy regarding duration of hospitalization, with no differences in other indicators observed [83]. According to another meta-analysis by Gan et. al. [84], including 600 patients reported in 8 studies, comparing two PPC groups (small tumor group (≤6 cm in diameter) and large tumor group (>6 cm in diameter)), laparoscopic adrenalectomy is a safe and effective approach for large adrenal PGL, and transabdominal laparoscopic adrenalectomy is better than retroperitoneal laparoscopic adrenalectomy [84].

In conclusion, surgery is the standard management for PPC; less invasive surgical approaches are used more and more frequently. Open surgery has been replaced by laparoscopic and robotic approaches, whereas total adrenalectomy has been replaced by cortex-sparing adrenalectomy. In our opinion, less invasive surgery is very useful for numerous patients, because it is associated with faster patient recovery compared to open surgery; however, this approach has some limitations. It does not allow for broad exposure and the digital tumor palpation that is needed in large, advanced tumors with vessel involvement. Therefore, surgical approaches should be selected for each patient individually. Regarding robot-assisted surgery, meta-analyses have shown that the robotic procedure was significantly better than laparoscopy with regard to intraoperative blood loss, duration of hospitalization, and conversion to open procedure, while postoperative morbidity and readmission rates were similar in both groups. Longer durations of operation, higher costs, and the absence of tactile feedback, which could inadvertently increase tumor manipulation and catecholamine release, are disadvantages of the robotic approach. In our opinion, further large, randomized prospective studies comparing laparoscopic and robotic adrenalectomy are needed. Concerning cortex-sparing adrenalectomy, it is recommended for patients with a high risk of bilateral adrenal PGL and a low risk of malignant behavior in order to avoid the long-term dependence on corticosteroids leading to various, adverse, drug-related effects. Cortex-sparing resection is not recommended for patients with SDHB PPCs due to a high risk of locoregional recurrence and/or metastasis. In our opinion, in such patients, a total adrenalectomy and not a cortex-sparing procedure, regardless of tumor size, should be performed to avoid locoregional recurrence and metastasis.

### 7.3. Genitourinary Sympathetic EAPGLs

Genitourinary sympathetic EAPGLs are infrequent tumors (<1% of all PGLs). The most common location is the urinary bladder (79.2%), followed by the urethra (12.7%), pelvis (4.9%), and ureter (3.2%). These tumors are typically manifested by micturition-induced attacks of headache and palpitations. Radical or partial cystectomy and transurethral removal are performed. Radical cystectomy is the preferable treatment, which is associated with the fact that in patients following transurethral resection, EAPGL recurrence is possible, because EAPGLs are not mucosal tumors [81].

### 7.4. Thoracic Sympathetic EAPGLs

Mediastinal and cardiac EAPGLs are very rare tumors arising from the para-aortic or para-vertebral sympathetic ganglia. These tumors are characterized by a poor response to radio-chemotherapy and therefore complete surgical resection is the standard of care [81,85]. A surgical approach depends on the tumor location within the mediastinum. Superior mediastinal EAPGLs can be resected by a transcervical approach. In tumors located more inferiorly, within the middle or posterior mediastinum, or that are intimately involved with the large vessels, a median sternotomy may be required [81,86]. Due to the highly vascular blood supply of mediastinal EAPGLs and invasion of the large vessels, the use of cardiopulmonary bypass may also be needed for the prevention of additional patient’s hemodynamic instability and possibility of complete resection [81,86,87,88]. Due to their difficult location and invasion of surrounding structures, complete tumor resection occurs only 77% of the time and is related to an 85% 15-year survival rate with a mean survival of 125 months [81,86,88]. Incomplete resection of mediastinal EAPGLs is related to a 60% 15-year survival rate with a mean survival of 71 months [81,86].

### 7.5. Head and Neck PGLs (HNPGLs)

The management of HNPGLs depends on their location, multifocality and genotype. Surgery is the optimal management choice. The goal of surgery is to achieve and prevent the injury of adjacent cranial nerves and vessels. Currently, mortality due to vascular complications is near 0% and it has been decreased from 30–40% previously. However, surgical morbidity secondary to cranial nerve injury is still significant, commonly in bilateral/multiple tumors. Therefore, despite the fact that surgery is the only treatment, alternative treatment strategies, including active surveillance and radiotherapy are considered [81,89]. A transcervical approach is most frequently used for HNPGLs, most commonly with carotid body tumors (CBT) and less frequently in vagal or sympathetic chain EAPGLs [81].

#### 7.5.1. Carotid Body PGL (CB PGL)

Surgery is the only radical management option for resectable CBT. Preservation of the internal and external carotid arteries is required. In a case of major arterial sacrifice due to local infiltration, artery bypass grafting and intraoperative monitoring of cerebral function are required. Resection is challenging and it is related to a > 15% complication rate regarding cranial nerves (X, superior laryngeal, XI, XII), sympathetic trunk palsies, and vascular complications [89]. Based on imaging, CBT is classified according to the Shamblin classification depending on internal and external carotid arteries (ICA and ECA) involvement. This classification is essential in preoperative preparation and operative planning [81,89,90]. The Shamblin classification, introduced in 1971 [90] can predict vascular complication risks based on tumor diameter and carotid artery involvement. Three classes (localized, partially wrapped, and wrapped) are distinguished in the Shamblin classification (Table 5). A perioperative stroke risk following CBT resection is frequently related to ICA manipulation, ligation, or reconstruction. Therefore, the tumor involvement of ipsilateral ICA, as well as the patency of the contralateral ICA, is the most important consideration in preoperative planning. Thus, in modified Shamblin classifications, the relationship between the tumor and ICA/ECA is precisely assessed based on imaging (Table 6 and Table 7) [89,91,92].

In patients with type III CBTs according to the Shamblin classification, due to a significant risk for ICA reconstruction or ligation, a preoperative balloon ICA occlusion investigation for evaluation of the cerebral blood supply via collateral vessels is required. Intraoperative endovascular intervention is considered in patients with large tumors in whom intraoperative distal ICA control is difficult. Total CBT removal is recommended in young and fit-for-surgery patients. CBT transarterial embolization is indicated before operation for a decrease in intraoperative blood loss. It is recommended that embolization be performed < 48 h prior to surgical procedures in order to prevent revascularization edema and vascular damage caused by hypoxia-induced inflammation and congestion of the vasa vasorum. It should be added that performing embolization before surgery is controversial, because embolization is related to a higher risk of cranial nerve damage. Moreover, blood loss or transfusion requirements were similar in patients with and without preoperative embolization. Preoperative embolization increases the cost of medical care and the risk of cerebral embolization [89]. Therefore, there is no consensus regarding the influence of preoperative embolization on the surgical outcomes of CBT resections. A meta-analysis by Abu-Ghanem et al. [93], including 470 patients reported in 15 studies, showed no significant difference in estimated blood loss, duration of operation, duration of hospitalization, risk of cranial nerve and vessel injury, and stroke between the embolization and non-embolization groups [93]. A correlation between Shamblin classification and the risk of perioperative stroke in patients undergoing surgery for CBT has been shown. A meta-analysis by Robertson et al. [34], including 4418 patients with 4743 CBT, showed a correlation between a 30-day stroke with the Shamblin classification in 544 patients. Shamblin I CBTs were related to a 1.89% stroke rate, increasing to 2.71% in Shamblin II CBTs and 3.99% in Shamblin III CBTs. In a study of 1075 patients, there was an association between cranial nerve injury rates and Shamblin’s classification with 3.76% in Shamblin I CBTs, 14.14% in Shamblin II CBTs, and 17.10% in Shamblin III CBTs, respectively [34]. These results confirm the importance of Shamblin classification in preoperative planning [34]. Similarly, another review by Wang et al. [94] confirmed these results and revealed that higher Shamblin CBT class tumors were related to a higher number of operative complications [94].

**Figure 4:**
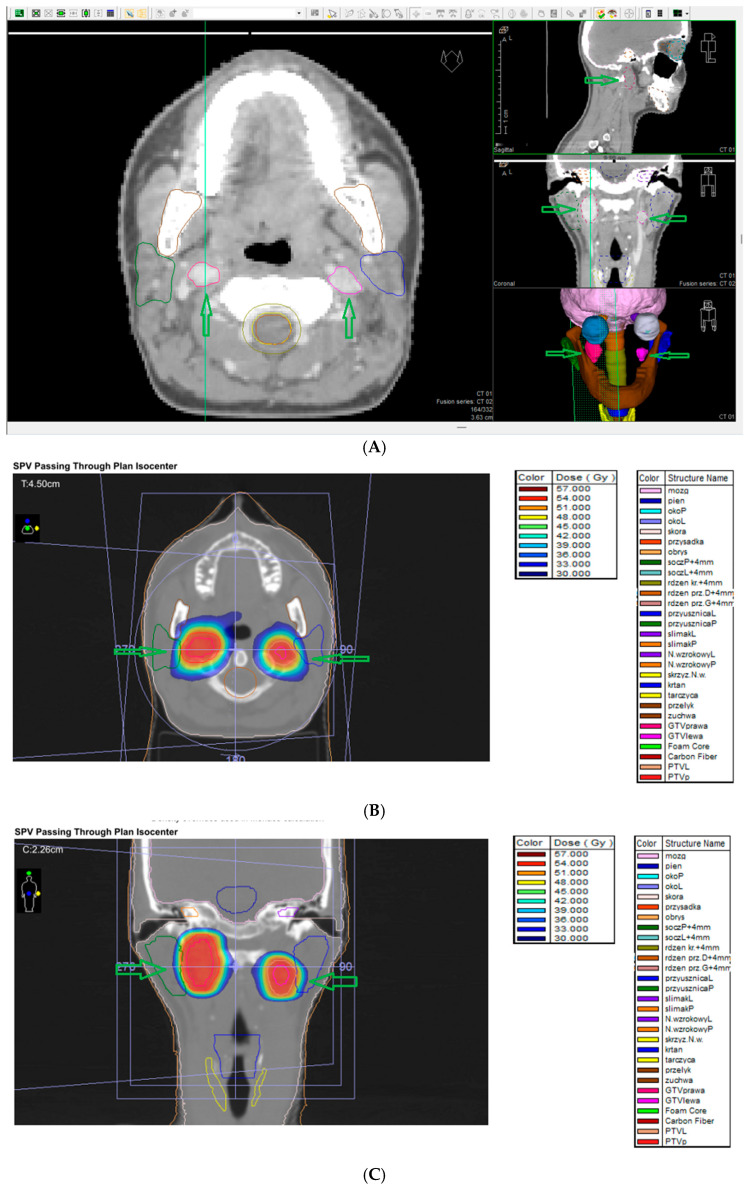
(**A**) Conventional radiotherapy for bilateral CBT in a 31-year-old white female patient with familial PPGL1 syndrome—SDHD C11X. CT for treatment planning; (**B**) conventional radiotherapy for bilateral CBT in a 31-year-old white female patient with familial PPGL1 syndrome—SDHD C11X. Dose distribution shown on the CT scan; and (**C**) conventional radiotherapy for bilateral CBT in a 31-year-old white female patient with familial PPGL1 syndrome—SDHD C11X. Dose distribution shown on the CT scan.

Complete tumor resection without permanent complications is the optimal management outcome; radiotherapy or chemotherapy are used in patients with Shamblin III tumors of diameter >5 cm [21]. Patients who are not fit for surgery, with numerous comorbidities, as well as older ages, may be qualified for other less invasive management options because of high postoperative morbidity rates [89]. Radiotherapy can be an alternative treatment method associated with a lower complication rate. However, the long-term results of radiotherapy are not described and can lead to other treatment-related complications typical for radiotherapy, including xerostomia, loss of hearing, cerebrovascular accidents due to vascular strictures, and malignant neoplasms [89]. Due to the abovementioned surgery- and radiotherapy-related complications, another alternative management option is active surveillance, called “wait and scan” (head and neck MRI is the examination of choice). It is possible in this patient group because of the frequent benignity and slow growth of these tumors. This management option prevents treatment-related morbidity, which is the most important consideration in patients with multiple and bilateral PGLs [89,95,96]. In Figure 4A–C, conventional radiotherapy images for bilateral CBT are presented.

In conclusion, the management of CBT is complex and difficult. Complete CBT removal is recommended in young and fit-for-surgery patients. CBT transarterial embolization before surgery is indicated for a decrease in intraoperative blood loss due to occlusion of the tumor feeding vessels. Taking into account that embolization is related to a higher risk of cranial nerve injuries as well as similar blood loss or transfusion requirements in patients with and without preoperative embolization according to some studies, its use is controversial. Moreover, preoperative embolization is related to an increased cost of medical care and increases the risk of cerebral embolization, which can lead to brain strokes. In our opinion, preoperative embolization should be considered before surgery only in large, advanced tumors with vessel involvement, but should not be a standard management option in all patients. The choice of an optimal therapeutic strategy for patients who are not candidates for surgery because of their age or comorbidities is very challenging. Radiotherapy is an alternative treatment method for some patients but is related to a risk of post-radiotherapy complications. In some patients with a very high perioperative risk, active observation should be considered.

#### 7.5.2. Tympano-Jugular PGL (TJ PGL)

Tympano-jugular paragangliomas (TJ PGLs) are uncommon tumors infiltrating and eroding bone and are closely related to critical structures, including cranial nerves and ICA, as well as intradural space [81,97]. Surgery of these tumors is related to a high morbidity rate caused by injury of involved cranial nerves. An infratemporal surgical approach is frequently used [81]. In some advanced tumors, ICA or facial nerve must be sacrificed [81]. Tympanic PGLs may be removed through a trans-canal endoscopic approach related to significantly lower morbidity [81,97]. Recently, subtotal resection has been introduced for these tumors. Subtotal resection is related to, lower postoperative morbidity (hearing loss, facial palsy and cranial nerve deficits, postoperative tinnitus and hearing loss) than traditional total tumor resection [81,97]. The other treatment strategies include microsurgery, preoperative embolization followed by surgical resection, fractionated external beam radiotherapy, and gamma knife radiosurgery [89]. A systematic review by Jansen et al. [98], including 18 studies involving 83 patients receiving radiotherapy and 299 receiving surgery, assessed the local control and complication rates in different management methods stratified by the Fisch classification [99]. The Fisch classification is presented in Table 8. The authors showed excellent post-surgery local control and a low risk of cranial nerve injury <1%. in class A and B tumors, 80–95% local control post-surgery (84% post-radiotherapy), and cranial nerve injury 71–76% (none post-radiotherapy) in C1-4 tumors. The authors did not show difference in treatment outcomes between tumors of different C class. Local control was 38–86% (98% post-radiotherapy) and cranial nerve injury rates were 67–100% (3% post-radiotherapy) in class C1-4De/Di tumors. In addition, lesser local control and cranial nerve damage rates were noted in C1-4DeDi tumors compared to C1-4De tumors. According to the authors, surgery seems to be a suitable treatment option for class A and B tumors, while in patients with class C and D tumors, initial surveillance should be considered. In patients with tumor growth (confirmed by imaging) or clinical tumor progression (result of early cranial nerves palsy), radiotherapy might be the better option because of lower complication rates and similar or better local control rates compared to surgery [89,98].

The second classification used in TJ PGLs is the Glasscock–Jackson classification that distinguishes two major tumor types: Glomus Tympanicum and Glomus jugular, with four different subgroups each. There is no difference between the timpano-jugular PGL and timpano-mastoid PGL in this classification. Both the glomus tympanicum and Glomus jugulare are classified in four classes that are presented in Table 9.

In TJ PGLs, the role of endovascular treatment is limited, because it is technically difficult to achieve complete lesion occlusion [89]. In these tumors, radiotherapy and active observation are alternative management options in order to prevent the surgery-related abovementioned complications and iatrogenic morbidity. According to Fancello et al. [97], when complete surgical TJ PGL resection is not possible, appropriate counseling and patient selection, including comprehensive tumor classification, should be performed before qualification for radiotherapy to control tumor progression, since surveillance may be optimal in selected patients. These authors, in a systematic review that included 9 studies, noted that the tumor progression rate in patients undergoing radiotherapy was 8.9%, while in the surveillance group, it was 12.9%. These results suggest innate, slow PGL growth. However, in the authors’ opinion, it is not possible to draw certain conclusions due to the heterogeneity of the analyzed studies [97].

In conclusion, the treatment of TJ PGL is very difficult due to its bone infiltration and erosion and a close relationship with critical structures, including cranial nerves and ICA, as well as intradural spaces. Therefore, surgical treatment of these tumors is associated with a high rate of injury of the involved cranial nerves. Tympanic PPGLs may be resected via a less invasive trans-canal endoscopic approach related to significantly lower morbidity. Subtotal tumor resection is related to lower complication rates secondary to cranial nerves deficits than traditional complete tumor resection. The other treatment strategies include microsurgery, preoperative embolization followed by surgical resection, fractionated external beam radiotherapy, gamma knife radiosurgery, and active observation. In our opinion, the choice of management should be individualized depending on local factors (such as tumor size and anatomical relationship to cranial nerves and vessels) as well as the patient’s general condition (such as age and comorbidities). In young patients without comorbidities, surgery is the optimal management option, while in patients with large, advanced tumors infiltrating vessels or cranial nerves, as well in patients not fit for surgery because of their general condition, other management options (such as radiotherapy and active observation) should be considered.

#### 7.5.3. Vagal Nerve Paraganglioma (VN PGL)

Vagal nerve paraganglioma (VN PGL) is the third most common HNPGL. The only treatment method is surgery. The aim of surgery is to completely remove the tumor while preserving the carotid artery, the jugular vein, and the cranial nerves as much as possible. According to the literature, in only 5–8% of patients can the vagal nerve be spared, without any procedure-related morbidities [21,102,103]. Numerous factors are important in the management of vagal nerve PGL, including the patient’s age and general condition, the tumor biological behavior and diameter, genetic results, bilateral and multicenter location, lower cranial nerve function, and a risk of postoperative morbidity. Taking into account that the most frequent behavior is benign (6–19% of tumors have malignant behavior, but with the highest malignancy rate among all HNPGLs) and the slow growth of the tumor, active observation is an alternative option of management in asymptomatic patients over 60 years of age. Surgery is needed in patients in whom the tumor is eroding the skull base or extending intracranially, or for the prevention of additional injury of the lower (IX, X, XI, XII) cranial nerves. Surgery is the optimal management option for young “fit for surgery” patients. In older patients with a higher surgical risk and growing tumors, radiotherapy is recommended [21,89,104,105].

#### 7.5.4. Laryngeal Paraganglioma (VN PGL)

Laryngeal PGL are not common and are typically slow-growing and non-functional tumors located in both the supraglottic and infraglottic larynx [89]. Surgery is the optimal management choice, including endoscopic removal, microlaryngoscopy with laser excision, and open resection via a transcervical, lateral thyrotomy, or laryngofissure approach. Although the endoscopic approach is successful for smaller tumors, it is related to a higher recurrence rate. Due to high vascularization and adhesion to vital structures, preoperative radiotherapy or embolization for vascularity reduction is recommended by some authors. The use of preoperative radiotherapy is controversial because these tumors are most frequently diagnosed after surgical resection [89,106].

#### 7.5.5. Multiple Head and Neck Paragangliomas

In patients with bilateral HNPGLs, an individualized decision regarding management should be made, depending on the patient’s age, comorbidities, tumor diameter and location and neurologic function [21]. The treatment of bilateral vagal or jugular PGLs is the most difficult and challenging, because it is associated with a high risk of postoperative morbidity and failure caused by nerve and vessel damage. Therefore, according to most recommendations, surgery is replaced by an active observation or radiotherapy in cases of neurological dysfunction [1,21]. An alternative option is surgical resection of one of the symptomatic tumors—starting with the largest one— and active observation of the other tumors in order to prevent severe postoperative complications such as bilateral vagal nerve palsy [1,13,21]. A 3D-reconstruction is useful in planning surgery for patients with multiple PGLs, because it shows the anatomical relationships between the tumors and the nerves and vessels, leading to a decrease in postoperative morbidities such as nerve and vessel damage [21].

### 7.6. Postoperative Surveillance

Following an immediate postoperative period, it is recommended that biochemical assessment should be performed within 4 to 6 weeks after surgery. Biochemical follow-up should be repeated at 6 months and 1 year after surgery. In patients with elevated catecholamine concentrations, imaging at 3 months postoperatively or at the time of its detection if detection was within that timeframe is recommended. In patients with metastatic disease, CT/MRI surveillance is recommended every 3–6 months for the first year, followed by every 6–12 months for follow-up. In all patients with PPGLs, long-term annual follow-up (for at least 10 years) with routine clinical and biochemical assessment in addition to imaging is strongly recommended. In selected patients with a higher risk of metastasis or recurrence, functional imaging, such as ^68^Ga-DOTATATE PET/CT, is recommended for follow-up surveillance in addition to anatomical imaging studies. In patients with a high risk of malignancy, recurrence, and hereditary syndromes, lifelong follow-up is recommended [81,85,107,108,109,110,111,112].

The overall 10-year recurrence is 13–15% and 30-year recurrence is 33%, respectively, and it is higher in patients with hereditary syndromes. The highest risk of recurrence or metastasis is in patients with genetic syndromes following partial cortical-sparing adrenalectomy (up to 7–10% at 3 years postoperatively to 10% and 38.5% for VHL and MEN2, at 10 years. The larger tumor size (>4.5 cm) is related to a significantly higher metastasis risk [81,113].

The overall mortality in patients with PPGLs has significantly decreased due to improvements in perioperative care, including preoperative adrenergic blockade, as well as improved anesthetic and surgical techniques, and postoperative surveillance. Torresan et al. [113] noted a 5-year survival rate of 97–99% at 5 years, and 97% at 20 years in patients with non-metastatic disease. According to the literature, in patients with metastatic PGLs, 5- and 10-year overall survival (OS) is 60–86% and 70–71%, respectively [81]. According to Jimenez et al. [114] overall survival is related to TNM staging, but it is not related to hereditary disease, including SDHB mutation. The authors noted that OS rates were related to AJCC TNM staging and were not worse in hereditary PGLs. The 10-year OS probabilities were 0.844 in patients in tumor staging I, 0.792 in staging II, 0.595 in staging III, and 0.221 for staging IV. The authors reported a significantly shorter OS in patients at stage IV compared to stages I-III of the disease [114]. According to the literature, location of a primary tumor (adrenal vs. extra-adrenal), larger tumor diameter, older patient’s age at the time of diagnosis, biochemical tumor activity, presence of synchronous metastasis, and presence of germline mutations (SDHB) were adverse factors for OS [81].

### 7.7. Metastatic Pheochromocytomas/Paragangliomas

**Figure 5:**
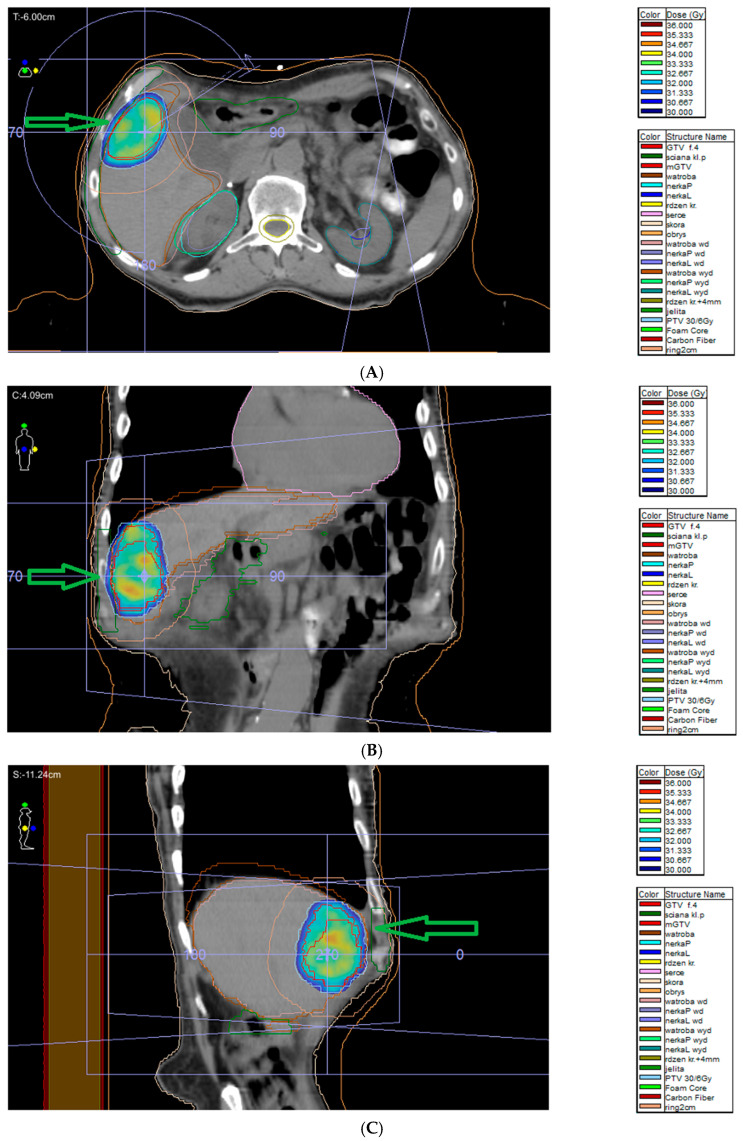
(**A**) Stereotactic radiotherapy for metastatic pheochromocytoma (liver metastasis) in a 73-year-old white male patient. Dose distribution shown on the CT scan; (**B**) stereotactic radiotherapy for metastatic pheochromocytoma (liver metastasis) in a 73-year-old white male patient. Dose distribution shown on the CT scan; and (**C**) stereotactic radiotherapy for metastatic pheochromocytoma (liver metastasis) in a 73-year-old white male patient. Dose distribution shown on the CT scan.

In metastatic disease, surgery is individualized, depending on clinical factors, following initial medical treatment of catecholamine-related symptoms. During debulking surgery, surgery should be performed if all tumors can be removed. However, debulking surgery could be considered only in patients with signs related to notable catecholamine secretion or mass effect. Treatment options depend on the patient’s general condition, progression severity, and the presence of catecholamine-related signs. Follow-up of treated patients is required, and the patient should be re-evaluated depending on treatment results. Local therapies include radiotherapy, radiofrequency ablation, cryoablation, microwave ablation, embolization, chemoembolization and palliative surgery ([^131^I]-MIBG, iodine-131 meta-iodobenzylguanidine; radioligand therapy (RLT); TKI, and tyrosine kinase inhibitor [68]). In Figure 5A–C, stereotactic radiotherapy for metastatic pheochromocytoma (liver metastasis) is presented.

### 7.8. Stereotactic Radiosurgery (SRS)

Stereotactic radiosurgery (SRS) has been introduced as a less invasive option compared to surgery management. It involves, as follows: a Gamma Knife [Elekta AB, Stockholm, Sweden] surgery [GKS], a linear accelerator (LINAC), and a CyberKnife [Accuray Inc., Sunnyvale, CA, USA]) [115]. In a meta-analysis by Ong et al. [115], including 23 studies (460 patients), 95% glomus jugulare control rate and 47% symptomatic improvement were shown. The median marginal dose used in the included studies varied from 14 to 20 Gy. The pooled tumor control rate was 95% over 47 (4–268) months [115]. Another meta-analysis by Dharnipragada et al. [116] including 19 studies (852 TJP patients) demonstrated similar rates of tumor recurrence in patients following stereotactic radiosurgery and surgical TJP resection. Radiation was related to a lower complication risk and lower morbidity; however, the reduction in tumor size was not significant. Based on these results, the authors concluded that radiosurgery was a reasonable management option in patients with minimal symptoms and with a high perioperative risk. According to the authors, microsurgical resection should be reserved for patients with lower cranial neuropathies or patients who have failed radiation treatment [116]. A meta-analysis by Ivan et al. [117], including 46 studies (869 GJT patients), compared the tumor control rates in patients undergoing SRS alone, gross total tumor resection (GTR), subtotal tumor resection (STR), and STR followed by SRS (STR + SRS). The authors demonstrated tumor control rates as follows: 95%, 86%, 69%, and 71% in SRS alone, GTR, STR, and STR + SRS groups, respectively. There was a significant difference in the mean rate of new-onset IX (9.7% and 38%), X (9.7% and 26%), and XI (12% and 40%) cranial nerve deficit between the SRS and GTR groups, respectively. The rates of the XII cranial nerve deficit were similar in both groups (8.7% and 18%, respectively) [117,118]. Another meta-analysis by Fatima et al. [119], including 37 studies involving 11,174 patients (1144 tumors) with glomus jugulare (GJT: 993, 86.9%), glomus tympanicum (GTT: 94, 8.2%), carotid body tumors (CBTs: 28, 2.4%), and glomus vagale (GVT: 16, 1.4%) treated with SRS definitively or adjuvantly, showed that surgery (gross tumor resection or subtotal tumor resection) was twice as likely to be related to transient or permanent deficits compared to SRS. Moreover, primary radiotherapy or SRS has a 78% more probability of being related to local tumor control compared to surgery alone [118,119].

SRS is suitable for HNPGLs of <3 cm in maximum dimension. An advantage of SRS is administration in one fraction; it is, thus, convenient. A single dose of ablative irradiation (12.5–15 Gy) to the tumor with a very steep dose falloff to minimally radiate the adjacent normal tissues is the aim [1].

### 7.9. External Beam Radiotherapy (ERBT) and Hypofractionated Stereotactic Radiotherapy (hSRT)

**Figure 6:**
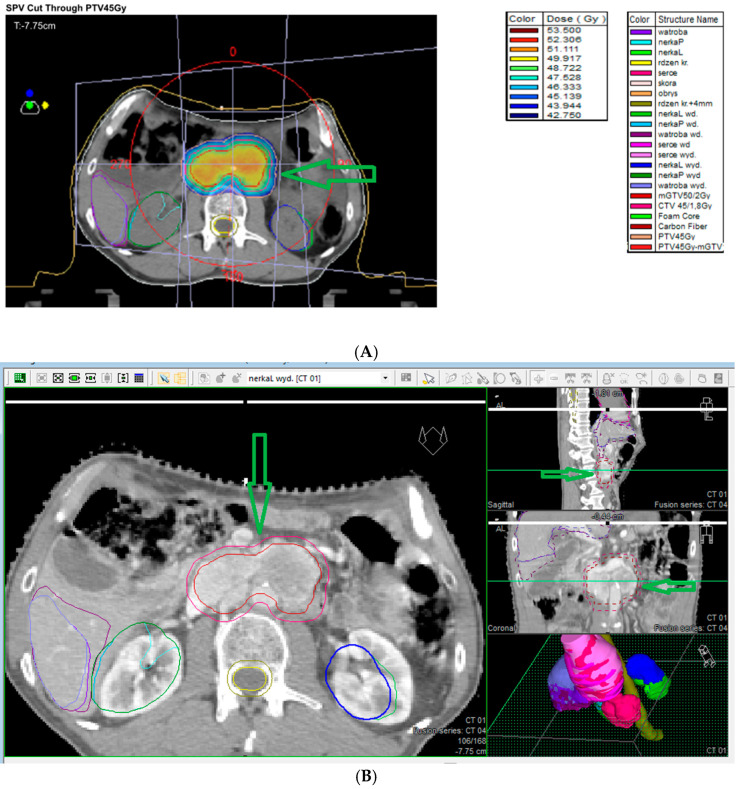
(**A**) Conventional radiotherapy for retroperitoneal paraganglioma in a 52-year-old white male patient. Dose distribution shown on the CT scan; and (**B**) conventional radiotherapy for retroperitoneal paraganglioma in a 52-year-old white male patient. CT for treatment planning.

Due to the surgical challenges of complex anatomy, high vascularity, and proximity to neurovascular structures, only subtotal excision is possible in numerous patients. External beam radiotherapy (ERBT) is used to treat partially resected PPGLs, as these tumors are radiosensitive. Radiotherapy is an alternative treatment option for patients not fit for surgery or SRS. EBRT, characterized by a high tumor control rate, is also recommended for PPGLs in patients not fit for surgery. The tumor control is good or better compared to surgery or SRS, and the risk of severe complications, including irradiation-induced malignancy, is <1%. The disadvantage of radiotherapy is that it requires a 5-week duration of treatment. Radiation is administered with an external beam usually employing 6 MV X-rays and an intensity-modulated radiotherapy technique with a 5 mm margin. The radiation dose is moderate (45 Gy in 25 fractions over 5 weeks) and significant morbidity is not common [1]. However, large fields must be covered for good tumor control. This can lead to brain parenchymal radiation necrosis, bone necrosis, and secondary malignancies. Moreover, other complications, such as headaches, hypopituitarism, mucositis, and cranial neuropathies, have been frequently reported following external beam radiotherapy [118]. Radiotherapy works by the induction of obliterative endarteritis, which fibroses surrounding blood vessels; however, due to the impact on surrounding tissues, the abovementioned complications are possible following EBRT [120]. An alternative radiation option to EBRT is hypofractionated stereotactic radiotherapy (hSRT) adopting the same technique as SRS but dividing the total dose into 3–5 fractions [120]. In Figure 6A,B, conventional radiotherapy for retroperitoneal paraganglioma is presented.

### 7.10. Systemic Therapy

Surgery is the treatment of choice for localized disease but should not be used for regionally advanced and metastatic PPGLs. In these cases, in functional tumors, symptomatic therapy including alpha-blockers, beta-blockers, catecholamine synthesis inhibitors, as well as chemotherapy, targeted therapy, SS analogs (SSAs), and radiometabolic therapy are used. In addition, locoregional procedures (cytoreductive surgery, external beam radiotherapy, arterial embolization, cryotherapy, RF ablation) are used [121].

#### 7.10.1. Chemotherapy

Chemotherapy is considered as the treatment of choice for patients without the significant uptake of radiotracers or rapid disease progression related to high neoplasm dynamics, or with severe uncontrolled signs caused by catecholamine secretion or mass effect [121]. The combination of cyclophosphamide, vincristine, and dacarbazine (CVD), introduced by Keiser et al. in 1985 for the treatment of malignant PPC, is the standard regimen, including SDHB patients [121,122,123,124,125]. A meta-analysis by Niemeijer et al. [124], including 4 studies involving 50 patients with malignant PPGL treated with a combination of CVD chemotherapy showed a partial response concerning tumor volume in about 37% of patients and a partial response concerning catecholamine excess in about 40% of patients [124]. In 2018, Jawed et al. [125] showed that a prolonged CVD regimen (median of 20.5 cycles) was particularly effective for 12 metastatic PPGL patients with SDHB mutations [121,125]. Another option for chemotherapy is temozolomide (TMZ) that is orally administered in contrast to intra-venous dacarbazine. It demonstrated similar efficacy in patients with SDHB mutations, as a first-line or second-line option [121].

#### 7.10.2. Targeted Therapy

##### Tyrosine Kinase Inhibitors [TKIs]

The use of TKIs is associated with the important role of angiogenesis in the pathogenesis of several PPGLs, including cluster 1 and some cluster 2 (RET-driven) tumors [121,126,127]. A meta-analysis by Zhou et al. [127], including 7 studies (160 patients), assessed the efficacy and safety of TKIs in metastatic PPGLs. The authors concluded that a partial response and disease control rate was achieved in more than 30% and up to 80% of patients, respectively [127]. The following TKIs are used in the targeted therapy of PPGLs: sunitinib (introduced as the first); pazopanib; axitinib; and cabozantinib. Due to the side effects of TKI treatment, including nausea, vomiting, diarrhea, skin rash, hypertension, and QTc prolongation, close monitoring of patients and adequate supportive therapy are essential during TKI therapy [121].

##### mTORC1 Inhibitor Everolimus

The use of mTORC1 inhibitor everolimus is associated with hyperactivation of kinase activity (RAS/RAF/ERK or PI3K/AKT/mTOR pathways) in patients with cluster 2 PPGLs [121].

##### Immunotherapy

In approximately of 30–50% of patients with metastatic PPGLs, germline SDHB mutations are presented. Tumor pseudohypoxia and necrosis, abnormal tumor cell replication, and angiogenesis are typical for this cluster. In addition, in many sporadic metastatic PPGLs, a similar molecular profile as that of SDHB *(+)* PPGLs in observed. These tumor features may prevent recognition of the neoplasm by the immune system. Pembrolizumab is a humanized IgG4κ monoclonal antibody that targets the programmed cell death ligands 1 and 2 (PDL-1; PDL-2) pathway, which is frequently taken over by cancer cells to avoid the immune system. A clinical trial on pembrolizumab (second phase) in patients with, including 11 patients with metastatic PPGLs, showed an objective response rate of 9% (95% CI: 0–41%), and clinical benefit rate of 73% (95% CI: 39–94%). In four patients, grade 3 adverse events related to pembrolizumab were reported. Grade 4 or 5 adverse events or a catecholamine crisis was not reported. The progression-free survival time was 5.7 months, and the median survival duration was 19 months [128,129].

##### Cold Somatostatin Analogs (Biotherapy)

“Cold” somatostatin analogs (SSAs), octreotide LAR, and lanreotide autogel are commonly used in the management of midgut and pancreatic NETs. Somatostatin receptor (SSTRs) overexpression (mainly SSTR2) has been reported in some PPGLs (particularly cluster 1 SDHx-related PGLs, which also show the best responses to peptide receptor radionuclide therapy (PRRT) [121]. A decrease in HIF-α expression in PPGL cells has been hypothesized. Therefore, these are notably efficient in PPGLs with Krebs cycle dysfunction, including SDHx-related PPGLs [121]. A multicenter study by Fischer et al. [130], including 202 patients with PPGLs (50% SSTR2 positive), analyzed the association between SSTR2 immunoreactivity and SDHB immunoreactivity, mutational status, and the clinical behavior of PPGLs. The authors reported that SSTR2 positivity was significantly related to SDHB- and SDHx-related PPGLs, with the strongest SSTR2 positivity in SDHB-related PPGLs. A significant association between SSTR2 expression and metastatic PPGLs not related to SDHB/SDHx mutation was reported. In metastatic tumors, the disease control rate in patients receiving SSTR-based radionuclide therapy was 67% and in patients receiving “cold” somatostatin analogs, the rate was 100%, respectively. The authors reported an association of SSTR2 expression with SDHB/SDHx mutations and metastatic disease, and confirmed a high disease control rate of somatostatin receptor-based therapies in metastatic PPGLs [129,130].

##### Radioligand Therapy [RLT]

According to the most recent guidelines, RLT can be an efficient treatment of choice in patients with slow to moderately progressive PPGLs and documented tumor uptake of the corresponding radioisotope. The reason for this treatment in PPGLs is the fact that it is known that in 80% metastatic tumors, higher expression of SSTR (notably SSTR2) or norepinephrine transporter compared to normal cells is reported [85,112,121]. SSTR2 and norepinephrine transporter overexpression plays a crucial role in the uptake of radiolabeled SSAs (^177^Lu or ^90^Y-SSAs) and of ^131^I-MIBG, which makes them a target for the therapy of metastatic tumors [121]. Functional imaging studies with ^123^I-MIBG and/or radiolabeled SSAs should be used as a qualification for this treatment [121]. Iodine-131 metaiodobenzylguanidine (I-131 MIBG) has been used for the diagnosis and treatment of malignant adrenal PGLs and EAPGLs since the 1980s [131]. Peptide receptor radionuclide therapy (PRRT) with ^177^Lu-DOTATATE and ^90^Y-DOTATOC showed efficacy in a metastatic setting of PPGLs where no standard therapies were established. A meta-analysis by Marretta et al. [132], including 12 studies (213 patients), showed pooled disease control rates of 0.83 (95% CI: 0.75–0.88) and 0.76 (95% CI: 0.56–0.89) for ^177^Lu- and ^90^Y-PRRT, respectively. The pooled disease control rate for PRRT was 0.81 (95% CI: 0.74–0.87). Based on these results, the authors concluded that these therapies could be considered in the multidisciplinary treatment of PPGLs as alternatives to I-131 MIBG and chemotherapy [132].

### 7.11. Ablation Therapy

A retrospective study by Kohlenberg [133], including 31 patients with metastatic PPGL with a total of 123 lesions treated using radiofrequency ablation (RFA) (n = 42), cryoablation (CRYO) (n = 23), and percutaneous ethanol injection (PEI) (n = 4), analyzed the results of ablative treatment. In 86% of the tumors, radiographic local control was reported; metastasis-related pain or symptoms related to catecholamine secretion were reported in 92% procedures. There were no complications in 67% of the procedures. Clavien–Dindo Grade I, II, IV, and V complications were reported in 14%, 14%, 2%, and 2% of the procedures, respectively. Based on these results, the authors concluded that in patients with metastatic PPGL, ablative therapy can be efficient in the local control and relief of signs [133].

## 8. Summary and Conclusions

PPGLs are infrequent neuroendocrine hypervascular tumors arising in various locations of the paraganglion system. They are divided into sympathetic (including pheochromocytomas and extraadrenal paragangliomas) and parasympathetic extraadrenal tumors. These tumors are usually benign and slow-growing; about 90% of them are located in the adrenal paraganglia (so-called pheochromocytomas). The extraadrenal tumors most frequently are located within the abdomen (85%), followed by the thorax (12%), and head and neck (3%). Approximately 25% of PPGLs are malignant (about 10% of adrenal PGLs and about 20% of EAPGLs), defined by the presence of distant metastases, which can be diagnosed with the primary tumor or even 20 years later. About 25% of PPGLs are related to germline mutations, which are risk factors for multifocal and metastatic disease [134].

The clinical presentation of PPGLs is very different and depends on the anatomic site. PPGLs are manifested frequently by the classic triad of diaphoresis, headache, and palpitations as well as hypertension; however, clinical presentation related to catecholamine/metanephrine secretion varies widely. HNPGLs are usually non-functional and are manifested by neck mass, headache, cough, hoarseness, dysphagia, otalgia, pulsative tinnitus, hearing loss, and lower (IX-XII) cranial nerves deficits. PPGL diagnostics involves laboratory investigations, as well as anatomical (MRI/CT) and functional imaging. Regarding laboratory diagnostics, 24 h urine-fractionated and plasma-free metanephrine tests are very sensitive (>90%) in PPGLs. The screening tests of choice are plasma metanephrines investigations due to the ease of sample collection and higher specificity than 24 h urine tests.

Surgery is the treatment of choice for locoregional disease. In abdominal PPGLs, open, laparoscopic, and robotic techniques are used depending on the tumor size, malignant potential, presence of germline mutations, and previous surgery. Transabdominal and retroperitoneal approaches are used. Surgery for HNPGLs is challenging due to close tumor relations to adjacent nerves and vascular structures. In order to decrease the risk of iatrogenic injuries, preoperative embolization is used; however, this is associated with a higher risk of embolization incidents that can lead to ischemic cerebral stroke. In patients who are not fit for surgery (mainly elderly patients with numerous comorbidities with slow-growing, stable tumors), active observation is recommended in order to prevent postoperative complications related to nerves and vessel damage. In functional PPGLs, medical blockade before any surgery or procedure is used in order to avoid a hypertensive crisis in patients with benign or malignant PPGLs. Alpha-adrenergic receptor blockers are commonly used. In selected patients, techniques less invasive than surgery may be used (stereotactic radiosurgery, and hypofractionated stereotactic radiotherapy (hSRT)). Currently, external beam radiotherapy (ERBT) is used less frequently due to its complications. In metastatic disease, systemic therapies, including chemotherapy, targeted therapy (tyrosine kinase inhibitors [TKIs], mTORC1 inhibitor everolimus, immunotherapy, cold somatostatin analogs (Biotherapy), radioligand therapy) are used. In addition, ablation therapy, including radiofrequency ablation (RFA), cryoablation (CRYO), and percutaneous ethanol injection (PEI) are used in metastatic disease as a palliative treatment. Overall mortality due to PPGLs has significantly decreased due to progress in PPGL diagnostics and treatment; the 5-year overall survival rate is more than 90%. As mentioned above, our paper is particularly focused on the current management practices for PPGLs. Therefore, a table (Table 10) summarizing recent meta-analyses regarding the various treatment methods used in PPGLs is presented. We decided to present a summary of meta-analyses as the most reliable studies according to evidence-based medicine (EBM).

To our knowledge this is the first such comprehensive review article regarding the etiopathogenesis, diagnostics, and current management of all PPGLs. Most of the published reviews regard a small subgroup of PPGLs, and there are no reviews summarizing current knowledge on all PPGLs in the literature. This review article summarizes current knowledge which can be helpful in the management of PPGL patients.

## 9. Future Directions

Further studies on molecular diagnostics of PPGLs are needed. Numerous gene mutations associated with PPGLs are already known; however, further studies to find other currently unknown gene mutations are required. Moreover, algorithms for performing genetic tests in patients with PPGLs are needed. According to Endocrine Society Clinical Practice Guidelines on PPGLs, germline gene testing is recommended for every patient with PPGL [46,150]. Currently, numerous gene mutations are still described in patients with PPGLs. Therefore, it should be precisely determined which gene mutations in which patient should be tested. Further studies to find sensitive and specific plasma biomarkers in non-functional PPGLs are required.

Currently, it is well known that surgery is the treatment of choice for locoregional disease. The indications for less invasive abdominal procedures (laparoscopic and robotic approaches), as well as cortex-sparing adrenalectomy, should be precisely determined and respective algorithms are needed. Surgery for HNPGLs is challenging due to close tumor relations to adjacent nerves and vascular structures. The indications for total gross resection, subtotal resection, endoscopic approaches, radiotherapy or active observation should be precisely determined. Regarding HNPGL surgery, the role of preoperative embolization and indications for this procedure should be precisely determined. Generally, separate guidelines for the management of all HNPGL types are needed. Regarding systemic therapy used in the metastatic disease, targeted therapy, including immunotherapy is still in progress; further investigations, including targeted molecular and genetic therapies, in this area are required. Cluster-specific therapy of metastatic PPGLs has not yet entered clinical routine practice, although the distinctive molecular pathology (including signaling pathways of specific cluster-related PPGLs) suggests that some therapeutics may be more effective than others in a particular cluster [127,151]. Therefore, this therapeutic direction needs further study.

## Figures and Tables

**Table 1:** Comparison of PASS and GAPP scores for the prediction of malignancy in PPGLs.

PASS (Points)	GAPP (Points)
Histologic pattern Large nests or diffuse growth (2)	Histologic pattern Zellballen (0) Large or irregular nests (1) Pseudorosettes (1)
Cellularity High (2)	Cellularity Low <150 cells/U (0) Moderate 15–200 cells/U (1) High >250 cells/U (2)
Necrosis Present (2)	Necrosis Absent (1) Present (2)
Invasion Vascular invasion (1) Capsular invasion (1)	Invasion (vascular or capsular) Absent (0) Present (1)
Mitosis Mitotic figures (more than 3/10 high-powered fields) (2) Atypical mitoses (2)	Ki67 proliferation index <1% (0) 1–3% (1) >3% (2)
Other features Extension to adipose tissue (2) Cell spindling (2) Cellular monotony (2) Nuclear pleomorphism (1) Nuclear hyperchromasia (1)	Catecholamine type Epinephrine elevated with or without norepinephrine (0) Norepinephrine and/or dopamine but without epinephrine (1) Nonsecreting (0)
Maximum Score = 20	Maximum Score = 10

GAPP, Grading system for Adrenal Pheochromocytoma and Paraganglioma; PASS, Pheochromocytoma of the Adrenal Gland Scaled Score; PPGL, pheochromocytoma/paraganglioma.

**Table 2:** Clinical manifestation of PPGLs.

Clinical Manifestation in Patients with Pheochromocytomas and Paragangliomas
Headache; Diaphoresis; Palpitations; Tachycardia; Hypertension (persistent, episodic, or relative to baseline); Syncope; Anxiety; Tremor; New-onset hyperglycemia (or worsening of controlled diabetes mellitus); Weight changes; No symptoms.

**Table 3:** Pheochromocytoma and paraganglioma locations in hereditary syndromes.

Gene	Syndrome Name	Head and Neck	Thoracic	Abdominal Adrenal (PCC)	Abdominal Extraadrenal	Malignancy Risk
SDHA	PGL5	30–<60%	<10%	30–<60%		<10%
SDHB	PGL4	10–<30%	10–<30%	10–<30%	30–<60%	30–<60%
SDHC	PGL3	90–100%	<10%	<10%	<10%	<10%
SDHD	PGL1	30–<60%	10–<30%	30–<60%	30–<60%	<10%
SDHAF2 (SDH5)	PGL2	90–100%	Never reported	Never reported	Never reported	Never reported
RET	MEN2	<10%	Never reported	90–100%	<10%	<10%
VHL	VHL	<10%	<10%	90–100%	10–<30%	<10%
NF1	NF1	Never reported	Never reported	90–100%	<10%	10–<30%
TMEM127	-	10–<30%	Never reported	90–100%	10–<30%	Never reported
MAX	-	Never reported	Never reported	90–100%	10–<30%	10–<30%

PGL, paraganglioma syndrome; SDHD, succinate dehydrogenase subunit D; q, short arm of a chromosome; AD, autosomal dominant; SDHAF2, succinate dehydrogenase complex assembly factor 2 gene; SDHC, succinate dehydrogenase subunit C; SDHB, succinate dehydrogenase subunit B; p, long arm of a chromosome; VHL, von Hippel–Lindau syndrome; TMEM127, transmembrane protein 127; SDHA, succinate dehydrogenase subunit A; MEN2, multiple endocrine neoplasia type 2; NF1, neurofibromatosis 1; MAX, MYC-associated factor X.

**Table 4:** The association between biochemical profiles and specific genetic mutations.

Gene Mutation	Molecular Cluster	Biochemical Profile
SDHx or VHL	Pseudohypoxia	Noradrenergic
SDHx (often SDHB)	Pseudohypoxia	Dopaminergic or Methoxytyramine
RET or NF1	Kinase-signaling	Adrenergic

NF1, neurofibromatosis 1; RET, rearranged during transfection; SDH, succinate dehydrogenase; VHL, von Hippel–Lindau.

**Table 5:** Shamblin classification [89,90].

Class	Type	Description	Degree of Resection Difficulty	Type of Resection
I	Localized	The tumor separates the ICA and ECA, adjacent to the bifurcation	Easy resection	Routine dissection
II	Partially wrapped	The tumor is adherent and partially surrounding the carotid arteries	More difficult resection	Sub-adventitial dissection
III	Wrapped	The tumor is completely encasing the carotid arteries	Very difficult resection and interrupting cerebral circulation is almost always inevitable	Partial or complete vascular resection

**Table 6:** Modified Shamblin classification (taking into account precise carotid arteries involvement) [89,90].

Class	Type	Description	Resection
I	Localized	Smaller tumors with minimal vessel involvement or the contact between CBT and ICA ≤ 180 degrees on MRI.	The tumor can be resected with minimal morbidity
II	Partially wrapped	Larger tumors with possible ICA vs. ECA involvement (≥180 degrees and ≤270 degrees of contact to ICA).	Resection is still deemed possible with a greater risk of morbidity.
III	Wrapped	Large tumors with circumferential involvement of the carotids or ≥270 degrees of contact between CBT and ICA.	Internal carotid artery reconstruction or ligation is predicted.

**Table 7:** Modified Shamblin classification (taking into account the tumor size) [91,92].

Class	Tumor Size	Description	Resection Difficulty
I	<4 cm	No involvement or invasion the carotid arteries	No difficulty
II	>4 cm	Partial involvement or invasion the carotid arteries	Difficult
IIIA	>4 cm	Close involvement or invasion of the carotid arteries	Difficult, requires repair, removal or replacement of the carotid artery
IIIB	Each size	Class I, II or III according to the original Shamblin classification with invasion of the carotid arteries	It is necessary to confirm the invasion of the vessel wall clinically and/or histopathologically

**Table 8:** Fisch classification [98,99,100].

Tumor Class	Tumor Location and Extension
A	Tumors that arise along the tympanic plexus on promontory.
B	Tumors with invasion of hypotympanum; cortical bone over jugular bulb intact.
C1	Tumors with erosion of carotid foramen.
C2	Tumors with destruction of carotid canal.
C3	Tumors with invasion of carotid canal; foramen lacerum intact.
C4	Tumors with invasion of foramen lacerum and cavernous sinus.
De De1 De2	Tumors with intracranial but extradural extension: up to 2 cm displacement; more than 2 cm displacement.
Di Di1 Di2	Tumors with intracranial and intradural extension: up to 2 cm intradural displacement; more than 2 cm intradural displacement.

**Table 9:** Glasscock–Jackson classification [21,101].

Glomus Tympanicum		Glomus Jugular	
I	Small mass limited to the promontory	I	Small tumor involving jugular bulb, middle ear, and mastoid
II	Tumor filling middle ear space	II	Tumor extending under internal auditory canal; may have intracranial canal extension (ICE)
III	Tumor filling middle ear and extending into the mastoid	III	Tumor extending into petrous apex; may have ICE
IV	Tumor filling middle ear, extending into the mastoid or through tympanic membrane to fill the external auditory canal; may extend anterior to the carotid.	IV	Tumor extending beyond petrous apex into clivus or infratemporal fossa; may have ICE

**Table 10:** Summary of the recent meta-analyses on current management practices for pheochromocytomas and paragangliomas.

Authors	Year	Number of Studies (Patients)	Aim of Meta-Analysis	Results/Conclusions
Gan et al. [83]	2022	26 studies (2985 patients)	Comparison of robotic-assisted and laparoscopic adrenalectomy for adrenal PGL	Robotic technique superior to conventional laparoscopy for blood loss,), hospitalization duration, and conversion to open. Similar duration of operation, complication and readmission rates Longer duration of operation in retroperitoneal robotic-assisted surgery compared to laparoscopic approach.
Gan et al. [84]	2023	8 studies (600 patients)	The role of laparoscopic adrenalectomy in treatment of large adrenal PGL (>6 cm)	Similar complication rate in laparoscopic approach in small and large PPC. Longer duration of operation, duration of hospitalization, greater blood loss, hypertension, hypotension, and conversion in large tumors. Transabdominal is superior to retroperitoneal LS.
Wang et al. [135]	2024	6 studies (658 patients)	Comparison of robotic-assisted and laparoscopic adrenalectomy for adrenal PGL	No differences in duration of operation, transfusion rate, conversion rate, complication rate, intraoperative max SBP, intraoperative min SBP between RA and LA. Less blood loss, a shorter duration of hospitalization in RA compared to LA.
Schiavone et al. [136]	2024	10 studies (1202 patients)	Comparison of total adrenalectomy and subtotal adrenalectomy for bilateral adrenal PGL	Less post-surgical primary adrenal insufficiency after subtotal adrenalectomy compared to total adrenalectomy. Higher postoperative recurrence rate after subtotal adrenalectomy compared to total adrenalectomy.
Wangs et al. [137]	2023	15 studies (3542 patients)	Comparison of preoperative α-blockade and no blockade for PPGL patients undergoing surgery	Prolonged hypotension and vasopressor usage following α-blockade compared ton no α-blockade. Similar intensive care unit admission, duration of operation, overall cardiovascular morbidity, and mortality in both groups.
Zawadzka et al. [138]	2023	25 studies (1444 patients)	Comparison of partial and total adrenalectomy in bilateral adrenal PGL	A lower risk of postoperative loss of adrenal hormone function and acute adrenal crisis following partial adrenalectomy compared to total adrenalectomy. A higher risk of postoperative PPC recurrence following partial adrenalectomy compared to total adrenalectomy. Similar risk of metastasis and overall mortality in both groups.
Gan et al. [139]	2022	10 studies (898 patients)	Comparison of minimally invasive adrenalectomy (MIA) with open adrenalectomy (OA) in patients with large adrenal tumors (≥5 cm)	MIA superior to OA for duration of hospitalization, drainage duration, and fasting duration estimated blood loss and transfusion. Similar duration of operation and complication rate in both groups.
Fu et al. [140]	2020	14 studies (626 patients)	Comparison of open surgery and laparoscopic surgery for PPC	Lower rates of intraoperative hemodynamic instability, less intraoperative blood loss, lower blood transfusion rates, earlier ambulation, and food intake, shorter drainage tube indwelling time and postoperative stay and lower overall complication rates in LS compared to OS. Similar duration of operation, postoperative blood pressure control, severe complications rate, postoperative hypotension or cardiovascular disease in both groups.
Li et al. [141]	2020	14 studies (743 patients)	Comparison of laparoscopic surgery versus open surgery for adrenal PGL	Smaller tumor size and higher body mass index in LS compared to OS. lower estimated blood loss, lower transfusion rate, lower hemodynamic instability, less postoperative complications, lower Clavien–Dindo score ≥3 complications, shorter return to diet time, and shorter duration of hospitalization in LS compared to OS.
Jiang et al. [142]	2020	4 studies (203 patients)	Comparison of of transperitoneal laparoscopic adrenalectomy with retroperitoneal laparoscopic adrenalectomy for adrenal PGL	Shorter duration of operation, less intraoperative blood loss, shorter duration of hospitalization in retroperitoneal laparoscopic adrenalectomy compared to transperitoneal one. Similar complication rate and incidence of hemodynamic crisis in both groups.
Schimmack et al. [143]	2020	4 studies (603 patients)	Comparison of preoperative α-blockade and no blockade for adrenal PGL patients undergoing surgery	Similar mortality, cardiovascular complications, mean maximal intraoperative systolic and diastolic BP, and mean maximal intraoperative heart rate in patients with or without α-blockade.
Hamidi et al. [144]	2017	20 studies (1338 patients)	Analysis of predictors for mortality rates in patients with metastatic PPGL	Higher mortality associated with male gender and synchronous metastases
Abu-Ghanem et al. [93]	2016	15 studies (470 patients)	Comparison of preoperative embolization and no preoperative embolization in patients undergoing surgery for CBT	Similar estimated blood loss, duration of operation, duration of hospitalization, risks of cranial nerve injury, vascular injury, and stroke in embolization and no-embolization groups.
Texakalidis et al. [145]	2019	25 studies (1326 patients)	Comparison of preoperative embolization and no preoperative embolization in patients undergoing surgery for CBT	Shorter duration of operation, lower intraoperative blood loss in embolization group compared to no-embolization group. Similar rates of cranial nerve injuries, stroke, transient ischemic attacks, duration of hospitalization in both groups.
Wang et al. [94]	2024	25 studies (1326 patients)	Analysis of characteristics, management and operative complications of CBT	Lower estimated blood loss and shorter duration of operation, higher rate of stroke in embolization group compared to no-embolization group. Higher Shamblin grade tumors associated with more operative complications. More frequent relevant family history and more symptoms in SDHx mutation-positive patients compared to patient without mutation.
Robertson et al. [34]	2019	104 studies (4418 patients)	Analysis of characteristics, management and operative complications of CBT	Correlation of Shamblin status with stroke: I CBT associated with a 1.89% stroke rate, 2.71% for Shamblin II CBT and 3.99% for Shamblin III tumors. Correlation of Shamblin status with CNI rates: 3.76% for Shamblin I CBT, 14.14% for Shamblin II, and 17.10% for Shamblin III CBT. Similar drainage loss, rate of neck hematoma, and re-exploration rate due to hematoma in embolization and no-embolization groups.
Koh et al. [146]	2024	43 studies (8849 patients)	Analysis of demographics, clinical characteristics, treatment methods, and outcomes of SDH-mutated HNPGLs	Correlation between SDHD mutations and multifocality. Correlation between SDHB mutations and distant metastases. No correlation between SDH-related mutations and gender, age, tumor size, and familial occurrences.
Napoli et al. [147]	2023	5 studies (245 patients)	Analysis of effectiveness of a preoperative embolization according to different Shamblin classes	Lower blood loss in embolization group compared to no-embolization group. Similar duration of operation in both groups.
Fatima et al. [119]	2021	37 studies (11174 patients)	Analysis of effectiveness of stereotactic radiosurgery HNPGL	Surgery more frequently related to transient or permanent deficits compared to SRS. No difference in local control depending upon the SRS technique. SRS in HNPGLs associated with good clinical and radiological outcome.
Jackson et al. [148]	2015	22 studies (578 patients)	Assessment of effects of preoperative embolization on CBT surgery	Less estimated blood loss and shorter duration of operation in embolization group compared to no-embolization group.
Ghanaati H et al. [149]	2024	19 studies (328 patients)	Assessment of effects of preoperative embolization on GJT surgery	Less estimated blood loss and shorter duration of operation in embolization group.
Ong et al. [115]	2022	23 studies (460 patients)	Assessment of stereotactic radiosurgery for GJTs	95% tumor control rate 95% and 47% symptomatic improvement following SRS that may be a suitable treatment modality for GJTs.
Dharnipragada et al. [116]	2023	19 studies (852 patients)	Comparison of radiosurgery and surgical resection for TJP	Similar in both groups—3.5% tumor growth rate following radiosurgery vs. 3.9% recurrence rate in surgery. Lower (7.6%) complication rate for radiosurgery compared to surgery (29.6%).
Ivan et al. [117]	2011	46 studies (869 GJT patients)	Comparison of recurrence and cranial neuropathy after subtotal resection (STR), gross-total resection (GTR), STR with adjuvant postoperative radiosurgery (STR+SRS), and stereotactic radiosurgery alone (SRS)	Complete response, partial response and stable disease of, respectively, 4% (95% CI: 1–15%), 37%(95% CI: 25–51%) and 14% (95% CI: 7–27%).
Niemeijer et al. [124]	2014	4 studies (50 patients)	Assessment of chemotherapy with CVD on tumor volume in patients with malignant PPGL	4% complete response, 37% partial response and 14% stable disease. 14% complete, 40% partial and 20% stable hormonal response.
Zhou et al. [127]	2023	7 studies (160 patients)	Assessment of efficacy and safety of TKIs in metastatic PPGLs	0.320 partial response, 0.520 stable disease, and 0.856 disease control rates. Progression-free survival 8.9 months.
Marretta et al. [132]	2023	12 studies (213 patients)	Assessment of peptide receptor radionuclide therapy (PRRT) with ^177^Lu-DOTATATE and ^90^Y-DOTATOC in the metastatic PPGLs	0.83 and 0.76 disease control rate and 0.76 for ^177^Lu- and ^90^Y-PRRT, respectively. 0.81 pooled disease control rate for PRRT.

RA, robot-assisted; SBP, systolic blood pressure; LS, laparoscopic surgery; OS, open surgery; CBT, carotid body tumor; HNPGL, head and neck paraganglioma; PGL, paraganglioma; SRS, stereotactic radiosurgery; GJT, glomus jugulare tumor; TJP, tympano-jugular paraganglioma; TKI, Tyrosine kinase inhibitors; NPC, nasopharyngeal cancer; CVD, cyclophosphamide, vincristine and dacarbazine; PRRT, peptide receptor radionuclide therapy.
